# Head and neck cancer patients’ preferences for individualized prognostic information: a focus group study

**DOI:** 10.1186/s12885-020-6554-8

**Published:** 2020-05-07

**Authors:** Arta Hoesseini, Emilie A. C. Dronkers, Aniel Sewnaik, Jose A. U. Hardillo, Robert J. Baatenburg de Jong, Marinella P. J. Offerman

**Affiliations:** Department of Otorhinolaryngology and Head and Neck Surgery, Erasmus MC Cancer Institute, Erasmus University Medical Center, Dr. Molewaterplein 40, 3015 GD Rotterdam, The Netherlands

**Keywords:** Prognosis, Life expectancy, Head and neck cancer, Focus groups, Qualitative research, Quality of life, Survival, Communication, Patient-centered care, Shared decision making

## Abstract

**Background:**

Head and Neck cancer (HNC) is characterized by significant mortality and morbidity. Treatment is often invasive and interferes with vital functions, resulting in a delicate balance between survival benefit and deterioration in quality of life (QoL). Therefore, including prognostic information during patient counseling can be of great importance. The first aim of this study was to explore HNC patients’ preferences for receiving prognostic information: both qualitative (general terms like “curable cancer”), and quantitative information (numbers, percentages). The second aim of this study was to explore patients’ views on “OncologIQ”, a prognostic model developed to estimate overall survival in newly diagnosed HNC patients.

**Methods:**

We conducted a single center qualitative study by organizing five focus groups with HNC patients (*n* = 21) and their caregivers (*n* = 19), categorized in: 1) small laryngeal carcinomas treated with radiotherapy or laser, 2) extensive oral cavity procedures, 3) total laryngectomy, 4) chemoradiation, 5) other treatments. The patients’ perspective was the main focus. The interview guide consisted of two main topics: life-expectancy and the prognostic model OncologIQ. All focus groups were recorded, transcribed and coded. Themes were derived using content analysis.

**Results:**

While all patients considered it somewhat to very important to receive information about their life-expectancy, only some of them wanted to receive quantitative information. Disclosing qualitative prognostic information like “the cancer is curable” would give enough reassurance for most patients. Overall, patients thought life-expectancy should not be discussed shortly after cancer diagnosis disclosure, as a certain time is needed to process the first shock. They had a stronger preference for receiving prognostic information in case of a poor prognosis. Prognostic information should also include information on the expected QoL. The pie chart was the most preferred chart for discussing survival rates.

**Conclusions:**

The participants found it important to receive information on their life-expectancy. While most patients were enough reassured by qualitative prognostic information, some wanted to receive quantitative information like OncologIQs’ estimates. A tailor-made approach is necessary to provide customized prognostic information. A clinical practice guideline was developed to support professionals in sharing prognostic information, aiming to improve shared decision making and patient-centered care.

## Background

Head and Neck cancer (HNC) is an aggressive type of cancer characterized by significant mortality and morbidity [1–4]. Treatment is often invasive and interferes with vital functions such as breathing, swallowing, and speech. In addition, patients often face psychosocial problems and experience body image dissatisfaction as a result of the mutilating procedures [2, 5]. On the one hand physicians aim for cure and prolonging life, while on the other hand they strive for optimization of quality of life (QoL). This often results in a delicate balance between survival benefit and the functional, and psychosocial disabilities a patient is willing to accept after treatment. Therefore adequate counseling of patients including prognostic information can be of great importance. Previous research focused on whether or not to disclose the prognosis [6]. More recently the focus has shifted more in-depth to what information to provide, and how to do this [6–8]. This is in line with the increased attention for shared decision making (SDM). Patients need to be well-informed before they can be actively involved in treatment decisions [9, 10]. As patients may not be able to make well-informed treatment decisions without understanding their prognosis, providing prognostic information is a key factor in SDM.

We recently published the results of a qualitative research, focusing on treatment discussions among HNC patients and their doctors. We found that in only 6% of the consultations doctors provided quantitative prognostic information, by discussing numbers, such as percentages. In 94% qualitative prognostic information was provided, by using words such as “curable” and “good prospect” [11]. The current study is the second step in our qualitative research by exploring HNC patients’ preferences and views on receiving prognostic information. Relatively little attention has been paid to this topic. Some cancer patients want to know everything, while others are overwhelmed by too much information. Furthermore, each patient group has its own characteristics and preferences. For example, patients with breast cancer are considered to have high information needs [12]. To our knowledge, there are no studies published that explore HNC patients’ views on receiving quantitative prognostic information. Therefore, research is needed on *what* these patients want to know about their prognosis and in which *manner* they wish this information to be conveyed to enable better counseling and patient-centered care.

**Fig. 1:**
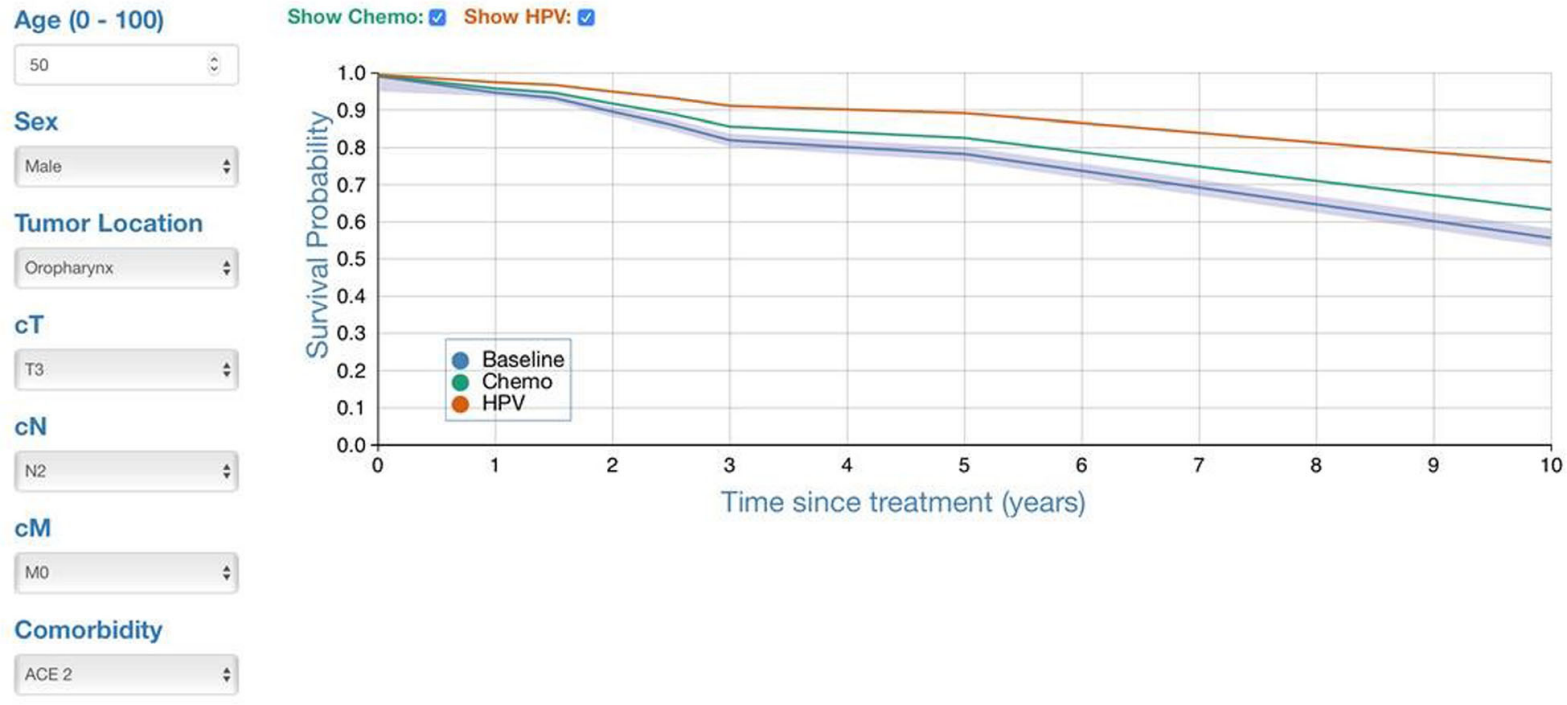
An example of OncologIQ’s estimates, as presented during the focus groups

Physicians are often unable to forecast an individual’s life-expectancy and tend to overestimate survival [13] (Hoesseini A, Offerman MPJ, van de Wall - Neecke BJ, Sewnaik A, Wieringa MH, Baatenburg de Jong RJ: Physicians’ clinical prediction of survival in head and neck cancer patients in the palliative phase, submitted). This can lead to concerns of being proved inaccurate and therefore reluctance to discuss the prognosis [14]. Survival rates of cancer are traditionally based on the TNM-classification of the tumor. These are however general estimates of a heterogeneous group of patients and not tailored to an individual’s prospect. Prognostic models that include patient specific predictors, like age and co-morbidity, could help doctors to provide a more personalized prognosis. Over the last years, an internally and externally validated prognostic model named “OncologIQ” has been developed. This model estimates the 1- to 10-year overall survival (OS) of patients with primary HNC, based on the average treatment effect [15–17]. Besides tumor location and TNM-classification, OncologIQ includes age, sex, and the Adult Comorbidity Evaluation 27 (ACE-27) as prognostic factors for OS (see also Fig. 1) [15–17]. The benefit of having a HPV-positive tumor or receiving chemotherapy were added by an adaptation method. This model could support doctors with prognostication during patient encounters, by providing more personalized estimates of the OS. However, it remains unclear if, how, and when this prognostic information should be shared with HNC patients? Furthermore, how should one visualize the individual survival estimates and in which manner should healthcare providers explain the results? While more prognostic models are developed, there is a dearth of evidence on the impact of the use of such models in clinical practice [18], and to what level patients appreciate and understand the information provided [19]. Our study fills this gap by exploring patients thoughts on OncologIQ.

The aim of the current study was to explore 1) HNC patients’ preferences for receiving prognostic information, 2) and their views on the prognostic model OncologIQ. By assessing patients’ views on these topics, we can optimize counseling between physicians and patients. In addition, a clinical practice guideline on how to use OncologIQ for individualized prognostic counseling was developed.

## Methods

We conducted a single center qualitative study by organizing five focus groups with HNC patients and their caregivers between December 2016 and February 2017. Methods and results are described using the Consolidated Criteria for Reporting Qualitative Research (COREQ) [20].

### Definition of prognosis

In this study we refer to the concept of prognosis from two different angles:

1. Qualitative information: general terms like “the cancer is curable”.
2. Quantitative information: numbers or percentages, like survival rates.

### Research team & reflexivity

The research team consisted of three investigators. M.P.J. Offerman (MO), PhD, is a psychologist and has several years of experience with focus group research. The second investigator, A. Hoesseini (AH), MD, is a physician, clinical epidemiologist, and PhD candidate. The third investigator, E.A.C. Dronkers (ED), MD, is also a physician, clinical epidemiologist, and PhD candidate. MO and AH conducted the focus groups. There was no relationship established with the participants prior to the beginning of the study. Treating physicians were not allowed to attend the focus groups, so participants would not feel reluctant to share their thoughts.

### Study design

This study was approved by the ethics committee of the Erasmus Medical Center (MEC-2013-052). After consulting experienced head and oncologists on how the groups should be selected, we divided patients in five common treatment groups, which is a reflection of the patient population we treat in our hospital: 1) small laryngeal carcinomas treated with radiotherapy or laser, 2) extensive oral cavity procedures, 3) total laryngectomy, 4) chemoradiation, 5) other treatments (local resection, neck dissection etc.). In this way, we selected patients who had a shared experience and thus were more likely to feel understood by each other. Based on the theory of social comparison [21], patients with a similar background feel more recognized and consequently less reluctant to share their thoughts.

**Fig. 2:**
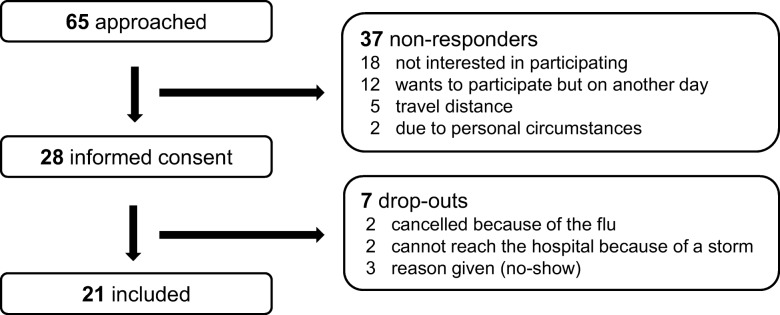
Patient selection procedure. Exclusion criteria were: aged 80 years or older; a carcinoma in situ; Korsakoff syndrome or dementia; severe alcohol and/or drugs abuse; possible recurrent or metastatic disease; recent hospitalization; simultaneous tumor outside of the head and neck region

Participants were consecutively selected by AH if they had undergone treatment for HNC in the Erasmus MC Cancer Institute, 6 to 18 months before selection. Patients were approached by telephone and information about the content and the working procedure of the focus groups was given. They were told that we wanted to learn from their experiences, with a main focus on how they had experienced the counseling by the healthcare providers. In order to limit selection bias, specific information on OncologIQ was not given in advance. Caregivers were encouraged to accompany patients. See Fig. 2 for the patient selection and exclusion criteria. Also, information on non-participants in shown in Fig. 2. In total 21 patients gave their informed consent and participated. All focus groups were held in the same conference room in the Erasmus MC Cancer Institute. Two volunteers were present during each focus group to welcome the patients. The volunteers did not know the patients and did not actively participate in the focus groups. Data were stored anonymously by study ID and were only accessible by the research team.

### Interview guide

An interview guide was made prior to the start of the focus groups (see Additional files 1 and 2). The main topics were 1) life-expectancy, and 2) the prognostic model OncologIQ. Each topic was first briefly introduced by AH and MO using a PowerPoint presentation (see Additional files 1 and 2). Subsequently closed-ended questions, using small cards, were answered by patients themselves. This enabled patients to react individually without being affected by the opinion of the other participants and their caregivers. The closed-ended questions were followed by open-ended questions to stimulate the group discussion, and caregivers were also encouraged to participate to a certain extent, as patients’ perspective was the main focus. Caregivers were invited as they are the main source of support for the patient and are often present during treatment decision consultations. Similar to these conversations, in the end the patient decides what kind of prognostic information is shared. OncologIQ was introduced only after the topic “life-expectancy” was thoroughly discussed. This order was deliberately chosen as we wanted to explore life-expectancy unbiased before introducing the prognostic tool. The model was demonstrated by showing a hypothetical patient with a different kind of tumor than the patients present in the focus group. The interview guide and presentation were adjusted once after the first focus group. In this first focus group we introduced quantitative terms like “5-year survival” directly after discussing life-expectancy in qualitative terms such as “curable”. This resulted in confusion among patients and caregivers. They interpreted the 5-year survival rate as “being told you only have five more years left to live” or confused it with the 5-year follow-up after the diagnosis. Therefore, we decided to introduce life-expectancy in qualitative terms more extensively before the break and introduce quantitative terms like 5-years survival after the break in the next focus groups. We also added one quantitative question on whether the physician should use a chart when explaining survival rates. After these adjustments no problems were encountered in focus group two until five, and therefore no further adjustments were made. All focus groups were digitally recorded. The mean duration of the focus groups was 2 h and 7 min. The focus groups were transcribed by AH and one of our volunteers.

### Data analysis

The grounded theory approach was used to analyze the data. This implies that the researcher moves back and forth between the population under study and analysis of the data, so that an explanatory theory evolves through an iterative process [22]. Two researchers (AH and MO) coded all transcripts and discussed the coding for each group until consensus was reached. Themes were derived from the coded data by AH and MO individually. These themes were discussed and if necessary rearranged, starting with one focus group, and adding the others one by one. When there was no agreement on the themes or on the matching of quotations with the themes, consensus was reached after an in-depth discussion. After discussing the fourth focus group, no new themes were identified and therefore data saturation occurred. The next step was verification of the results by the third researcher (ED). She was given parts of coded transcripts and was asked to match them with the identified themes, and if deemed necessary suggest new themes or codes. No new themes were identified by ED, however some (sub) themes were rearranged. Finally, one quotation per (sub) theme was jointly chosen to include in the results section. NVivo 12 was used to manage the data. The participants did not provide feedback on the findings.

## Results

### Participants

Table 1 shows an overview of the number of patients and caregivers in each focus group, and patient characteristics. In total 17 patients (81%) were accompanied by their caregiver(s). In 15/17 of the cases (88%) this was a partner. One patient took a sibling with her and one patient was accompanied by both his partner and two children. Education level was categorized according to the International Standard Classification of Education [23, 24]. Patients’ age and sex were similar to national HNC data gathered in the Netherlands Cancer Registry (NCR) by the Netherlands Comprehensive Cancer Organization (IKNL) [25]. Patients education level was more or less similar to a recent study among 2189 consecutive HNC patients in our tertiary center (Hoesseini A, van Leeuwen N, Offerman MPJ, Zhang J, Dronkers EAC, Sewnaik A, Lingsma, HF, Baatenburg de Jong, RJ: Predicting survival in head and neck cancer: external validation and update of the prognostic model OncologIQ in 2189 patients, submitted). This did not apply to marital status: while in the latter study 28% of patients were single, in the focus groups only 10% were.

**Table 1:** (a) Number of participants and (b) patient characteristics

Focus groups	Patients	Caregivers
1. small laryngeal carcinomas treated with radiotherapy / laser	6 (28.6%)	6 (31.6%)
2. extensive oral cavity surgical procedures	2 (9.5%)	2 (10.5%)
3. total laryngectomy	4 (19.0%)	6 (31.6%)
4. chemoradiation	5 23.8%)	3 (15.8%)
5. other treatments^a^	4 (19.0%)	2 (10.5%)
Total no. of participants per focus group (%)^b^	21 (100%)	19 (100%)
Patient characteristics	No. (%) / median (Q1-Q3)	
Age, years	65.0 (53.5–68.5)	
Age range, years	33–78	
Sex		
male	12 (57.1%)	
female	9 (42.9%)	
Tumor localization		
larynx	9 (42.9%)	
hypopharynx	2 (9.5%)	
oral cavity	3 (14.3%)	
oropharynx	6 (28.6%)	
unknown primary	1 (4.8%)	
Tumor stage		
I	5 (23.8%)	
II	3 (14.3%)	
III	5 (23.8%)	
IVa	7 (33.3%)	
IVb	1 (4.8%)	
Marital status		
married / durable relationship	19 (90.5%)	
single	2 (9.5%)	
Education level		
lower (primary education or less / lower secondary)	7 (33.3%)	
intermediate (upper secondary / post-secondary non-tertiary)	9 (42.9%)	
tertiary (short cycle tertiary / bachelor / master / doctoral)	4 (19.0%)	
missing	1	
Median time between end of treatment and participation in the focus group (Q1 – Q3)	47 weeks (35–64)	

^a^ For example neck dissection or local resection

^b^Two patients were treated for cancer recurrence by a total laryngectomy, the remaining were treated for a primary head and neck tumor

#### Life-expectancy

**Fig. 3:**
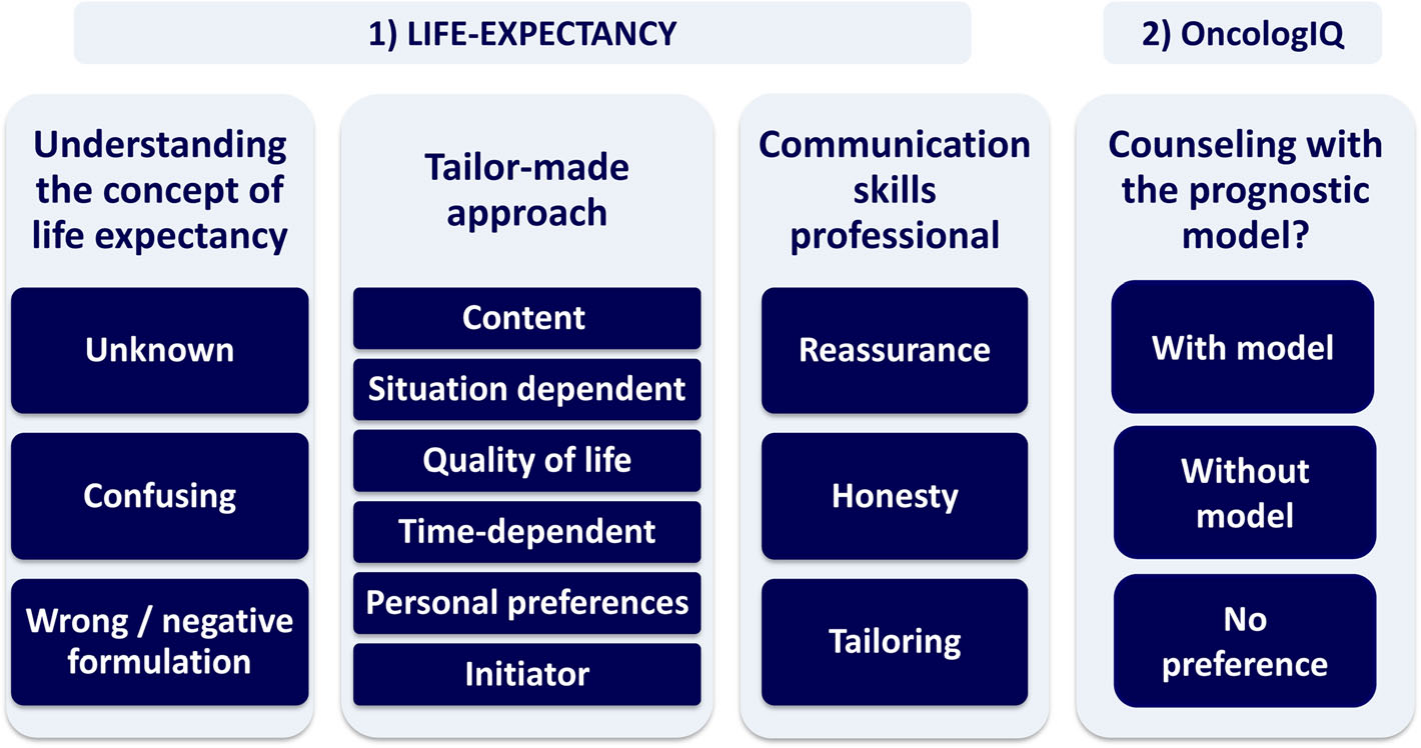
Code trees of themes and subthemes derived from the topics 1) life-expectancy and 2) the prognostic model OncologIQ

After the introduction of the main topic life-expectancy, we first asked patients the closed-ended question: To what extent do you think it is important to receive information about your life expectancy? (4-point Likert-scale, “not at all important” to “very important”, see also attachment 1). 62% of patients answered “very important”, the remaining eight (38%) answered “somewhat important”. Hereafter, open-ended questions were asked (see interview guide) to stimulate the group discussion. From the transcripts of these discussions in total three themes and 12 subthemes were derived (see Fig. 3 for the code tree and Table 2 for the contents).

**Table 2:** Explanation of (sub)themes and quotations**,** derived from the focus group discussions on topic 1) life-expectancy

Theme	Subtheme	Subcategory	Quotation^a^
**Understanding the concept of life expectancy** *What are patients’ views on the concept of prognosis, life-expectancy and 5-year survival rates?*	**1. Unknown**. Participants are not familiar with the concept life expectancy.		*I have never heard of the 5-year survival rate*. *(pt 3, f4)*
	**2. Confusing.** Participants don’t understand the different terms that are used alternately. This can be confusing.		*But what is actually meant by life expectancy? Do they mean survival chances, cure or life expectancy after treatment? (pt 2, f4) Or quality of life? (pt 1, f4)*
	**3. Wrong / negative formulation**. The 5-year survival term sounds negative. When talking about survival rates it should be emphasized that we are talking about chances, not certainties.		*It really should be said differently, but I do not know how… When you get home you only hear ‘five years, five years’. (pt 5, f1)*
**Tailor-made approach** *How should a professional provide customized prognostic information?*	**1. Content**. Prognostic information can be divided in 1) qualitative information: general terms without numbers or percentages, like “the cancer is curable”, and 2) quantitative information: numbers or percentages, like months, years or survival rates. All patients wanted to receive information in general terms. However, quantitative information was not desired by all patients. Some felt empowered by prognostic information expressed in numbers or percentages, and others were in doubt or did not want to receive quantitative information at all.	**1) qualitative information desired**	*If you say ‘well treatable’ I do not think that life expectancy is important. Well treatable is well treatable. Therefore that means the end result is also good. In that case I do not need to hear a percentage. (pt 6, f1)*
		**2) quantitative information…**	*I want to know what my chances are and find the percentages important. If you say ‘it is 3%’, it becomes somewhat more difficult. If I would hear 80% then I would think ‘all right, I'm definitely going to make it’. (pt 5, f4)*
		**… desirable**	
		**… value unclear**	*I find it somewhat difficult. You can say ‘I am part of the 70%’, but it could also be that you’re part of the 30% (…) What do you gain from those percentages? How can I tell you there is a 100% chance you will not be hit by a tram when you cross the road? (caregiver 1, f5).*
		**… not desired**	*I can imagine that someone just doesn’t want to know. Apart from the fact that the percentage says nothing, I can imagine that you do not want to hear it. If I would be in this situation again, I would not ask and say ’I don’t need to know’ (pt 1, f2).*
	**2. Situation dependent**. The need for quantitative prognostic information depends on the situation. In case of a poor prognosis patients have a strong preference for receiving quantitative prognostic information, while in case of a relative good prognosis patients are equally divided between wanting or not wanting to receive this information.	**1) good prognosis**	*In the case that positive results are expected, the doctor does not need to procrastinate and should just tell me. (pt 2, f1)*
		**2) poor prognosis**	*Suppose the cancer spreads and they say ‘there is no more treatment possible, if you want to know, you have six months left’. Then I think it is important to know. (pt 4, f1)*
	**3. Quality of life**. Prognostic information alone is not enough. Also information on the expected quality of life, with or without treatment, should be provided.		*You have to have a life. (pt 2, f3) At least a certain level of quality. (…) And you are always going to push your boundaries. You start with radiation and say ‘if the larynx goes, it is over for me’. You keep pushing that boundary, since an individual wants to stay alive. (caregiver 3, f3) Up to a certain limit. (caregiver 4, f3)*
	**4. Time-dependent**. If patients want to know more about their life-expectancy, for example survival rates, when should we discuss this? Overall, patients think this should not be discussed shortly after receiving the cancer diagnosis, because receiving the diagnosis is already an incredibly stressful event that first needs to be processed.		*At the time when I was at the doctor and heard I had a tumor, that information would be too much for me. (pt 4, f4)*
	**5. Personal preferences**. It depends on personal preferences whether a patients wants to receive prognostic information.		*First of all there are two patients groups. Some patients don’t want to know anything and say ‘just treat me and I’ll see’. Others want to know everything. (caregiver 4, f1)*
	**6. Initiator**. Who should take the initiative? How do you find out which patients want prognostic information, and what kind of information? Some patients will take the lead, while others aren’t capable or don’t want to, as they trust the doctor to do the right thing being the expert.		*I think the first step is that the patient says ’yes I want to know’ or ‘I don’t want to know’ and that he or she is also the one to say ‘I want or do not want the family to know’. (caregiver 1, f5)*
**Communication skills professional** *A customized approach requires certain communication skills of professionals.* *Which communication skills should a physician use when discussing the prognosis?*	**1. Reassurance**. Reassuring the patient and giving hope.		*You all want to hear: ‘everything will be alright’. Although I know that’s not realistic. (pt 2, f5)*
	**2. Honesty**. Being honest while providing prognostic information.		*To me it should be very clear. Just how it is and then I can see for myself what I will do with it. (pt 5, f1) Yes for me too. Do not sugar coat it and tell it straightforward, so I know what I am in for. (pt 2, f1)*
	**3. Tailoring**. Tailor prognostic information after exploring patients’ needs and preferences or decide not to share prognostic information at all when a patient isn’t ready for it.		*I think the doctor needs to look carefully at the patient. Can the patient handle the news at that time? (pt 1, f4) This means that you have a little more time with the doctor and that the doctor needs to know more of the personal history of the patient. (pt 2, f4)*

^a^*pt* patient, *f* focus group

#### The prognostic model OncologIQ

Table 3 gives an overview of the themes that were derived from the discussions on OncologIQ (see also Fig. 3 for the code tree). In addition, several recommendations were shared. Table 4 shows several visual formats of communication and patients’ preferences for the selected charts. The pie chart was the most preferred chart. All patients in focus group two until five (*n* = 15) preferred the combination of verbal explanation of survival rates and a visual presentation with a chart, over a verbal explanation solely. This was deemed easier to understand.

**Table 3:** (a) Explanation of (sub)themes, (b) recommendations and quotations, derived from the focus group discussions on topic 2) prognostic model OncologIQ

Theme	Subtheme	Quotations
**Counseling with the prognostic model?** *How do patients feel and think about counseling with OncologIQ?*	**With model**. Some patients want to be counselled with the prognostic model. They think it gives a clear overview of their survival chances, and provides a personal estimate of their survival rates.	*It makes it more personal I think. It applies more to you personally. (caregiver 2, f3)*
	**Without model**. Some patients don’t want to be counselled with the model. They find it too confronting, or just don’t feel the need to receive counselling with a prognostic model. Others think the model doesn’t include enough prognostic factors yet.	*If I’m part of the big group, I have more alternative possibilities.(pt 1, f5)*
	**No preference**. Some patients don’t have a specific preference, as they see both advantages and disadvantages of receiving prognostics information with a model.	*I sit on the fence a little. I think it is more confronting, but also somewhat more realistic. It is close to home and that can be frightening. So I am not sure whether I want it like that. (pt 4, f5)*
Recommendations		Quotations
**Add additional prognostic factors**, in order to make the prediction more individualized.		*I actually think it’s pretty unreliable. You should fill in many more things, like does the patient smoke, drink, and exercise? (pt 2, f4)*
**Add treatment modalities** if possible.		*Can you add radiotherapy in this model? (caregiver 1, f2)*
Include **quality of life** as an **outcome** in the model.		*This model says nothing about the quality of life. (caregiver 3, f3)*
Provide **structural information** to make sure every patient is informed about the possibility to discuss the individual prognosis with OncologIQ.		*People should be able to indicate in advance whether they want to know this or not. (pt 4, f5)*
This prognostic information should be given by **someone else than the physician**, as the participants thought this task would be too time-consuming and stressful for the physician. They opted to trust this task to a specialized nurse. In addition, one caregiver suggested to integrate this in our Healthcare Monitor.		*I think it's too much for a doctor. You become a doctor to help patients, but to really get to know the human psyche is something else. (caregiver 2, f5)*
Take **concerns about the health insurance** into account. In three focus groups caregivers shared their concerns about hypothetical consequences for the health insurance.		*Then the premium will increase. (caregiver 2, f3)*
**Show and explain all variables** that are included in OncologIQ. This enables patients to understand which variables are used to calculate their prediction.		*I think you should show the variables. This enables you to see what the prediction is based on. (pt 3, f3)*
**Use the 5-year survival rate**. When discussing survival rates, participants prefer using the 5-year survival rates instead of 1- or 10-year survival rates, unless the individual patient prefers otherwise.		
Create the possibility to view OncologIQ in a **patient portal**.

**Table 4:**
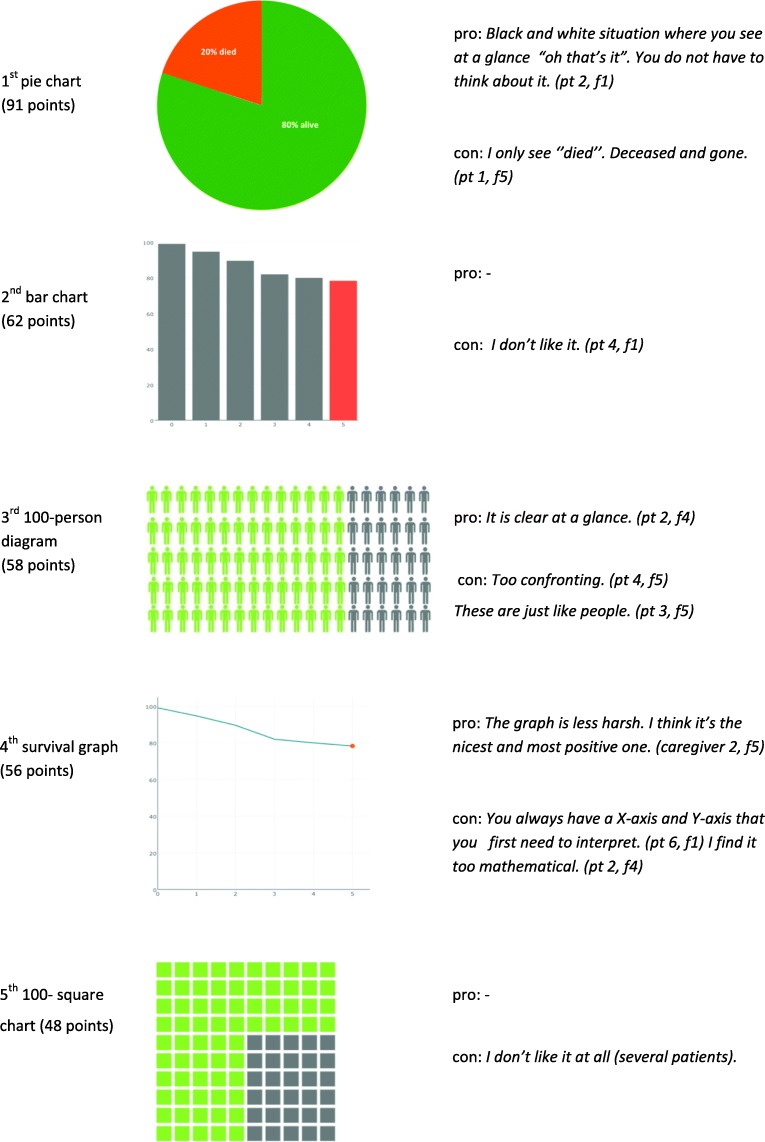
Visual formats of communication: chart preferences and patient quotations. Patients were asked which figure they would prefer when talking about life-expectancy*

*First choice nomination resulted in five points, last choice nomination in one point. In total 315 points were divided. Figure 2 until 5 also included captions with the ‘% died’ versus ‘% survive’, and if applicable captions of the x- and y-axis (not shown in this table)

## Discussion

### Life-expectancy

#### Understanding the concept & using a tailor-made approach

To our knowledge, this is the first study offering in-depth understanding of HNC patients’ preferences for disclosure of prognostic information, and the use of a prognostic model during treatment decision consultations.

While all patients considered it somewhat to very important to receive information about their life-expectancy, only some of them wanted to receive this in a specific quantitative manner, like 5-year survival rates. This is in line with previous research among patients with advanced or incurable cancer [26, 27]. The majority of patients wanted to receive prognostic information from their doctor in general terms, like ‘*your cancer can be well treated’.* This kind of qualitative information would give these patients enough reassurance for the first moment. Even though doctors generally use the concept *5-year survival rate*, participants often did not understand this concept or confused it with other terms, for example chances of cure, and thought it had a negative connotation. Overall, little is known about patients’ awareness, and understanding of prognosis [6]. Previous research stressed that in some cases cancer patients misunderstand or fail to absorb the information given, cannot recall the status of their disease and often overestimate their survival chances [6, 28–30]. The need for receiving prognostic information was dependent on different circumstances. This means that sharing prognostic information requires a tailor-made approach. Patients had a stronger preference for quantitative information like months or survival rates, in the hypothetical case of cancer recurrence and/or a poor prognosis. This kind of information would enable them to weigh whether undergoing a second treatment to prolong survival would be worth the ‘costs’. Prognostic information is not a standalone concept according to patients and caregivers. Patients also expressed the need for information about their expected QoL, since this would be of significant importance in the decision-making process. Fried et al. asked 226 patients with a limited life expectancy whether they would choose a treatment with survival, but with severe functional or cognitive impairment. 74.4% of patients answered they would not accept severe functional impairment and 88.8% would not accept cognitive impairment, and thus rather face death [31]. However, more recent research by Blanchard et al. among HNC patients showed that they overall prioritize survival over functional endpoints [32]. Although we did not explicitly ask patients to prioritize survival and QoL, they did however mention that at a certain point the survival benefits would not weigh against the deterioration in QoL. On the other hand they mentioned that patients are prone to keep pushing their boundaries, and increasingly accept functional limitations in order to stay alive.

In case patients want to receive quantitative information, what would be the right timing to share this? Our focus group results suggest that the right timing and phasing are of key importance. It seems that life-expectancy should be best discussed after the conversation in which the cancer diagnosis is given. According to most patients and their caregivers, it would be too stressful to discuss this all at once.

Several patients addressed that it depends on personal preferences whether a patient wants to receive prognostic information. While on the one hand some patients gain an increased sense of control by receiving more information about their disease and prognosis, others want to receive very little information. The latter group often wants the doctor to take control and is not interested in the details on treatment or prognosis. Receiving unwanted prognostic information could destroy hope and therefore patients’ needs should be explored beforehand [33], instead of bluntly confronting them with unwanted information. Who should take the initiative in exploring prognostic information needs? While some patients will take the lead, others aren’t capable or don’t want to. Therefore, according to the participants, the healthcare provider should be the one to introduce the topic, while the patient is given the opportunity to decide whether he or she wants to receive the information. This is in agreement with a qualitative research among advanced cancer patients: most patients and caregivers in this study said a physician should offer to discuss the prognosis, if the option to decline the information was also provided [34].

#### Communication skills professional

According to our participants, doctors should be honest while discussing the prognosis without taking away hope, and tailor prognostic information after exploring patients’ needs. The importance of being realistic and honest while maintaining hope is also identified in previous literature on patients with advanced or incurable cancer [35–38]. For example, Kutner et al. found that while 100% of patients in their survey wanted honesty from clinicians, 91% also wanted them to be optimistic [35]. Balancing between honesty while disclosing prognosis and maintaining hope can be a challenging task for healthcare providers [37, 39].

### The prognostic model OncologIQ

After fully exploring patients thoughts and believes on the topic life-expectancy, the prognostic model OncologIQ was introduced. Some patients would appreciate counseling with OncologIQ as they thought it was clear and more personalized, while others were in doubt. Some patients didn’t want counseling with OncologIQ at all because of the need to maintain some ambiguity about the future. This need to maintain ambiguity about outcomes, is also identified in previous research among advanced or incurable cancer patients [27, 33, 36]. Ambiguity could help to maintain hope and avoids a blunt confrontation with the facts. Participants shared several recommendations to improve the model. In three focus groups caregivers were concerned that the monthly health insurance premium would rise, if the insurance companies would also have access to an individuals’ prognostic estimate. Questions on this topic should be considered when using a prognostic model for counseling.

#### Visual formats of communication

Prognosis can be presented in various formats. While previous research showed that most persons find numbers and 100-person diagrams easiest to understand [40, 41], the HNC patients in this study preferred the pie chart. The pie chart was a favorite because they thought it was clear at a glance (see Table 4) and less confronting than some of the other formats. The 100-person diagram was considered too confronting by both patients and caregivers. This is in line with previous research that explored this by using a 100-faces diagram [41]. In addition, Davey et al. stated that the survival graph was considered negative, since it showed the constantly increasing mortality. In the current study, patients’ thoughts on the survival graph were also mostly negative. They found it too mathematical, since one must first must interpret the X- and Y-axis. Davey et al. also tested cancer patients’ understanding of the survival graph: only six out of 26 patients correctly interpreted the graph [41]. Furthermore, we assessed that the included patients’ preferred to combine verbal explanation with visual prognostic information over a verbal explanation solely. This is also reported in previous research on this topic [42]. Furthermore, it remains unclear as to what extent patients understand the uncertainty around prognostic models’ estimates [43]. Presenting data uncertainty is difficult and there is no consensus in literature about the optimal way to communicate different types of uncertainty [43, 44].

#### Practice implications: a guideline for individualized prognostic counseling

**Fig. 4:**
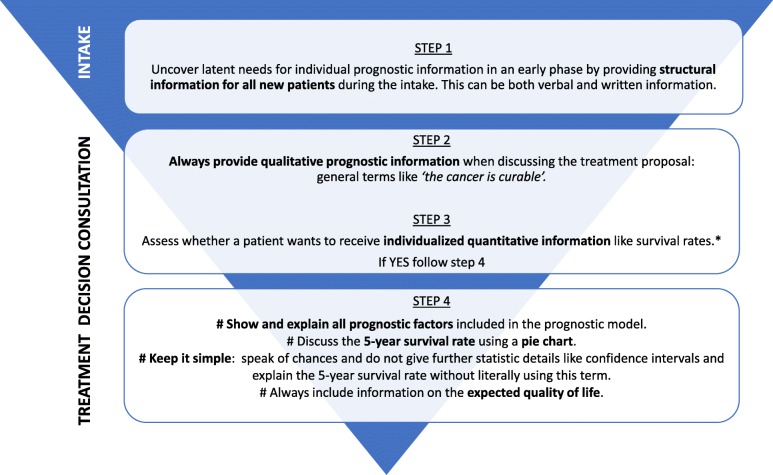
Clinical practice guideline for individualized prognostic counseling. *Keep the following in mind: do not to discuss life-expectancy in the same consult in which the cancer diagnosis is discussed but hereafter

OncologIQ could take away physicians reluctance to discuss the prognosis and reduce ambiguity in case of conflicting opinions among healthcare professionals by providing individual estimates. Previous research showed physicians’ willingness to use prognostic models in end-of-life care, aiming to improve prognostic confidence [14]. It also enabled physicians’ to take a more directive role in specific cases where the expected prognosis significantly differs from patients’ expectations, and it reduced ambiguity in case of conflicting opinions about prognosis among colleagues [14]. Based on the results of this focus groups study, especially the recommendations discussed in Table 3, a clinical practice guideline was developed that includes basic steps for sharing individualized prognostic information (see Fig. 4). While our earlier published guideline for professional communication focuses on general aspects of sharing prognostic information with HNC patients [11], this guideline specifically focuses on how to share the information provided by the prognostic model OncologIQ. It could also be used for other similar prognostic models in HNC. Since the term “5-year survival rate” seemed to confuse patients and caregivers, we recommend not to use it literally. We asked patients which survival period would be most appropriate if a patient wants quantitative prognostic information. Most patients preferred five years, as they deemed two years “too short” and 10 years “too far ahead”.

#### Strengths and limitations

One must first listen to patients’ preferences and needs, to be able to provide patient-centered care. The use of a qualitative methodology provided us with rich data on HNC patients’ preferences on these vital but unexplored topics. However, it is difficult to make assumptions on its generalizability. This study focused on patients with HNC in the curative setting. Since each setting has its own concerns, the generalizability of these results to the incurable setting is not desirable. Also, our results may be different in other, non-Western, cultures or countries. A certain selection bias may have occurred as the included patients are willing to participate in a focus group with other patients and caregivers. In addition, while almost one third of the patient population in our center is single, only 10% of patients in the focus group were. The presence of family members or other caregivers adds complexity to prognostic discussions since they may have different information needs [45]. However, we purposely chose to include caregivers in the focus groups, as they are also present during the treatment decision consultation.

#### Future perspectives

The results of the current study have been used to improve OncologIQ. Recently, the prognostic model has been updated (Hoesseini A, van Leeuwen N, Offerman MPJ, Zhang J, Dronkers EAC, Sewnaik A, Lingsma, HF, Baatenburg de Jong, RJ: Predicting survival in head and neck cancer: external validation and update of the prognostic model OncologIQ in 2189 patients, submitted). In the first place because the original model was based on outdated data as the survival of HNC patients has improved in the past years [46]. The second aim of the update was to test whether adding new prognostic factors would improve model performance, as recommended during the focus groups. Also, a visual format for patients has been developed, including a pie chart of the 5-year survival rate. The updated model can be found on www.oncologiq.nl. The next step will be to evaluate the clinical impact of OncologIQ in a prospective clinical trial. The primary outcome of this trial is decisional conflict among HNC patients who are counselled with and without the model during treatment decision consultations. The effect of the use of OncologIQ in our multidisciplinary tumor board meetings is also recently assessed in a pilot study.

A future aim would be to develop a prognostic model that includes both survival and QoL for HNC patients. Despite not addressing this future prospective during the focus groups, several patients stressed the importance of combining both survival and QoL, rather than focusing solely on survival. Due to the implementation of our Healthcare Monitor we will be able to meet this need soon (Dronkers EA, Baatenburg de Jong RJ, van der Poel EF, Sewnaik A, Offerman MP: Implementation of a standardized value based clinical support system ‘Healthcare Monitor’ for routine symptom monitoring of head and neck oncology patients, submitted). With this monitor we are collecting electronically patient reported outcomes (ePRO) on physical and psychosocial functioning since 2013, from intake until the last follow-up visit. In the first place this is done to improve patient care and counseling, although these data could also be used for research purposes.

## Conclusions

This study is first in examining HNC patients’ preferences for disclosure of prognostic information, and the use of a prognostic model. Overall, the findings of the current study highlight the importance of exploring patients’ thoughts and needs, in order to enhance patient-centered care. The participants found it important to receive information on their life-expectancy. While disclosing prognostic information in general terms like “the cancer is curable” gave enough reassurance for most patients, some also wanted numerical information like OncologIQ’s prognostic estimates. A tailor-made approach is necessary to provide this prognostic information in a customized manner. A clinical practice guideline was developed to support the healthcare professional in sharing individualized prognostic information, aiming to improve shared decision making.

## Supplementary information

**Additional file 1:** Material S1. Interview guide: overview of the topics and corresponding questions.

**Additional file2:** Material S2. PowerPoint presentation that was used during the focus groups.

## Data Availability

The datasets generated and/or analyzed during the current study are not publicly available due to the qualitative content of the dataset. The full dataset could contain information that might compromise research participants’ privacy and/or their conditions of consent. The data that support the findings of this study may be available on reasonable request from the corresponding author [AH].

## Abbreviations

COREQ: Consolidated Criteria for Reporting Qualitative Research
ePRO: Electronically Patient Reported Outcomes
HNC: Head and Neck cancer
QoL: Quality of Life
SDM: Shared Decision Making
