# Supplementary material for: Head and neck cancer patients’ preferences for individualized prognostic information: a focus group study
Source: BMC Cancer. 2020 May 7;20:399. doi: 10.1186/s12885-020-6554-8 (PMC7203788; doi:10.1186/s12885-020-6554-8)
Supplement: Supplementary file 1 — Additional file 1: Material S1. Interview guide: overview of the topics and corresponding questions. [file 12885_2020_6554_MOESM1_ESM.docx]

**Supplementary material**

Interview guide: overview of the topics and corresponding questions.

| Topics | **Answer type** |  |
| --- | --- | --- |
| Treatment decision consultation (warm-up topic) |  |  |
| 1. What do you think is a good treatment proposal?  2. To what extent do you want to be involved by your doctor when it comes to treatment choices? | open-ended  multiple choice* | *A: patient and doctor should decide together*  *B: the patient decides*  *C: the doctor decides* |
| Life-expectancy (main topic 1) |  |  |
| 1. To what extent do you think it is important to receive information about your life expectancy?  2. Do you think that life-expectancy should be discussed with each patient?  3. Should the doctor share survival rates with the patient? | multiple choice*    open-ended  open-ended | *4-point Likert-scale: ‘’not at all important’’ to ‘’very important’’* |
| The prognostic model OncologIQ (main topic 2) |  |  |
| 1. Which view would you prefer? (see table 4)  2. What would your preference be: 1) only verbal explanation of the percentages or 2) verbal explanation and showing a chart?  3. What do you think of this model? (see figure 1)  4. What would you change?  5. Do you think you would be better informed with the information in this model?  6. Do you think that the information in this model would be appropriate for everyone? | multiple choice*  multiple choice*  open-ended  open-ended  open-ended  open-ended | *see table 7: all patients were asked to choose a preferred order* |

* All multiple choice questions were answered by patients themselves. During open-ended questions caregivers were encouraged to participate in the group discussion to a certain extend as the patients’ perspective was the main focus.
