# Supplementary material for: Head and neck cancer patients’ preferences for individualized prognostic information: a focus group study
Source: BMC Cancer. 2020 May 7;20:399. doi: 10.1186/s12885-020-6554-8 (PMC7203788; doi:10.1186/s12885-020-6554-8)
Supplement: Supplementary file 2 — Additional file2: Material S2. PowerPoint presentation that was used during the focus groups. [file 12885_2020_6554_MOESM2_ESM.pptx]

## Slide 1
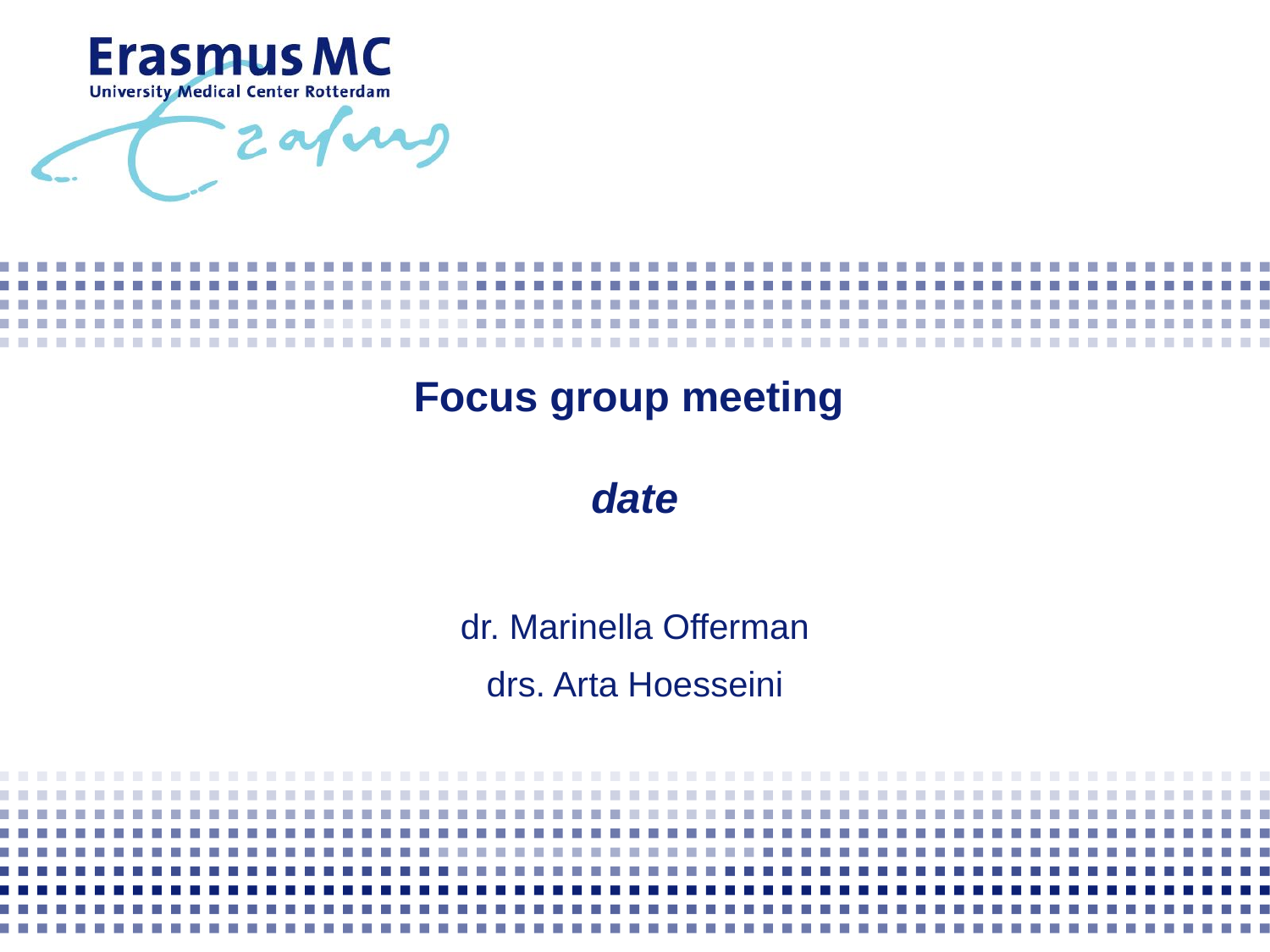

# Focus group meeting date
dr. Marinella Offerman
drs. Arta Hoesseini

## Slide 2
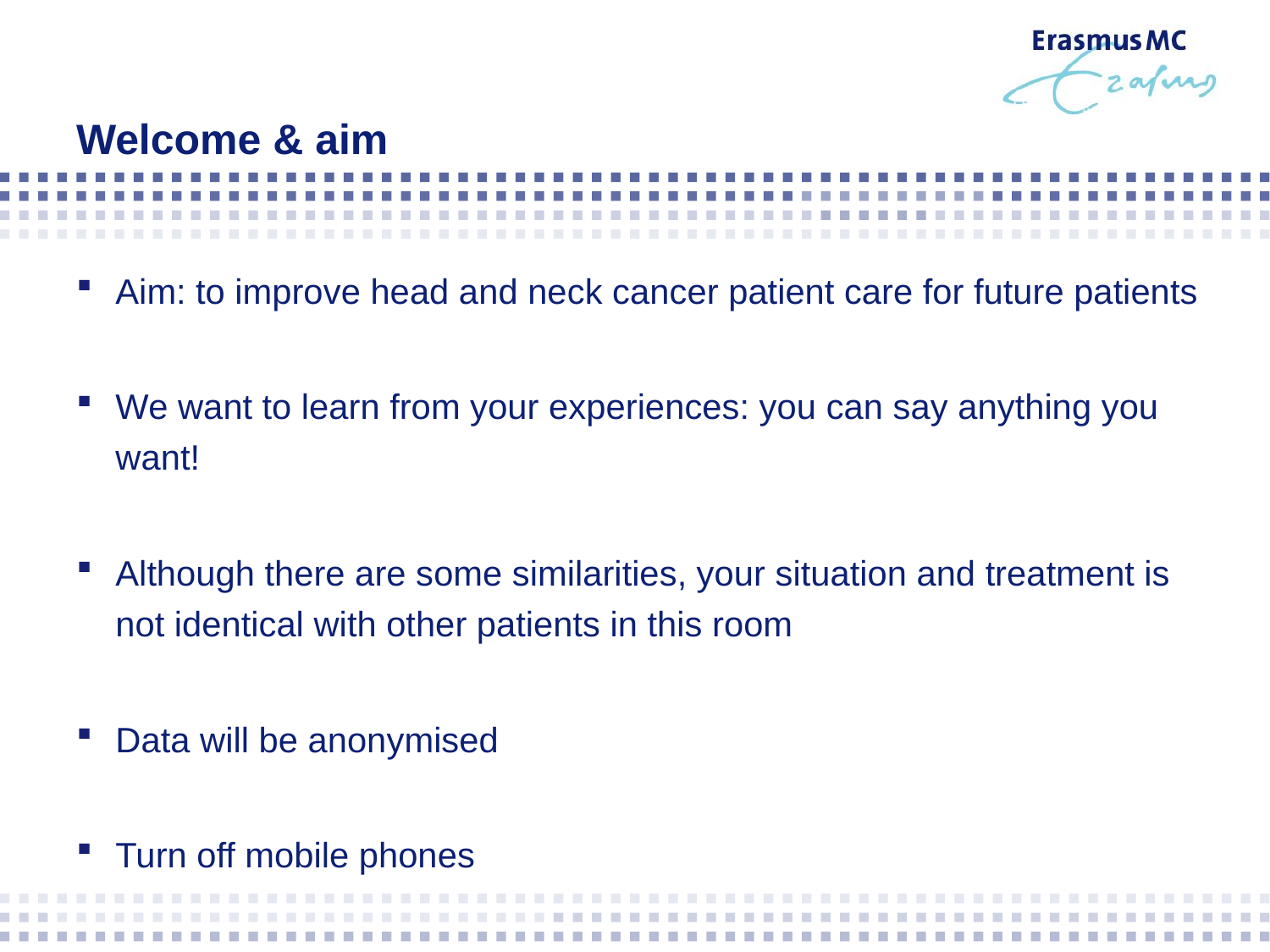

# Welcome & aim
Aim: to improve head and neck cancer patient care for future patients
We want to learn from your experiences: you can say anything you want!
Although there are some similarities, your situation and treatment is not identical with other patients in this room
Data will be anonymised
Turn off mobile phones

## Slide 3
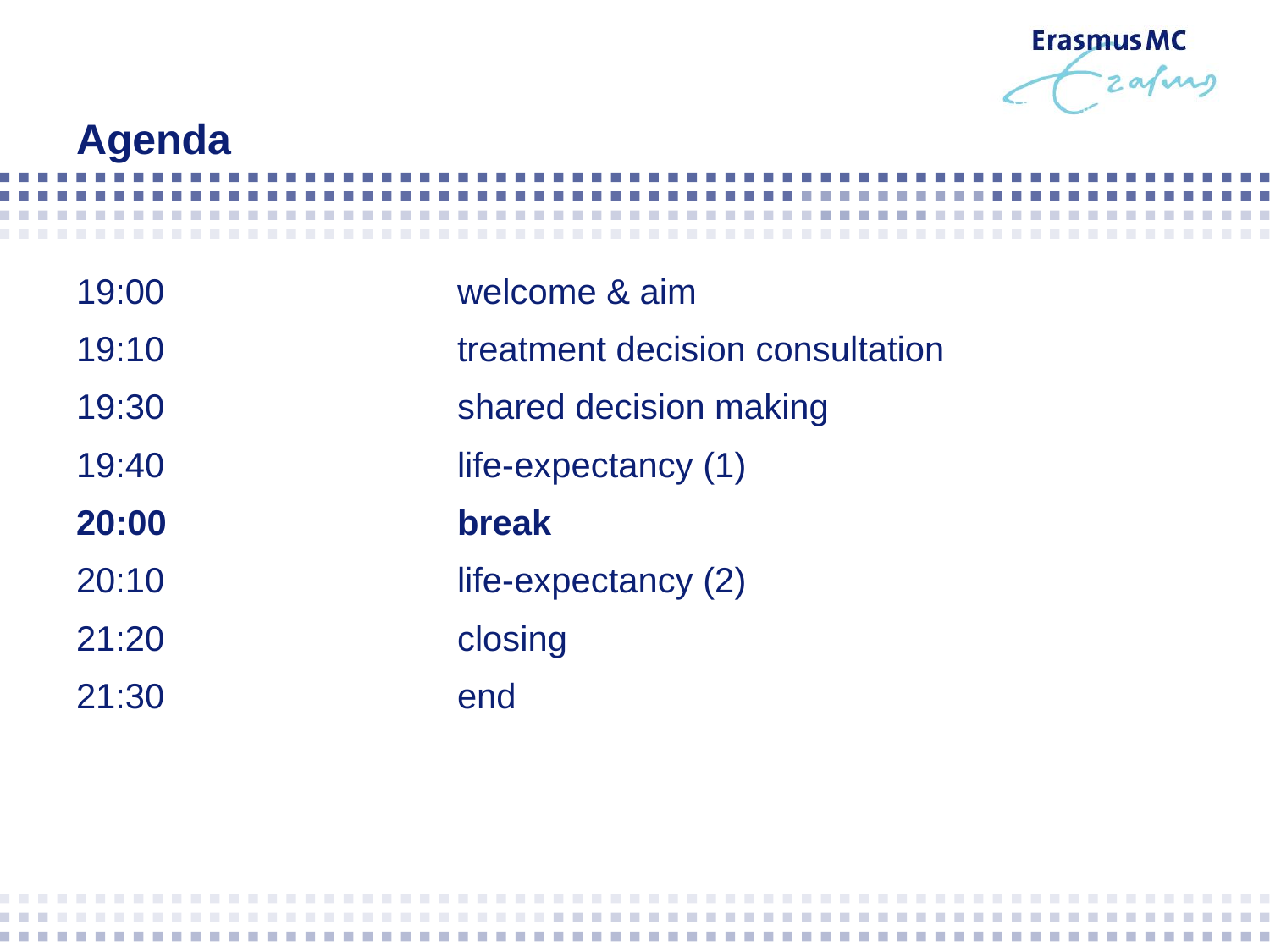

# Agenda
19:00			welcome & aim
19:10			treatment decision consultation
19:30			shared decision making
19:40			life-expectancy (1)
20:00			break
20:10			life-expectancy (2)
21:20			closing
21:30 			end

## Slide 4
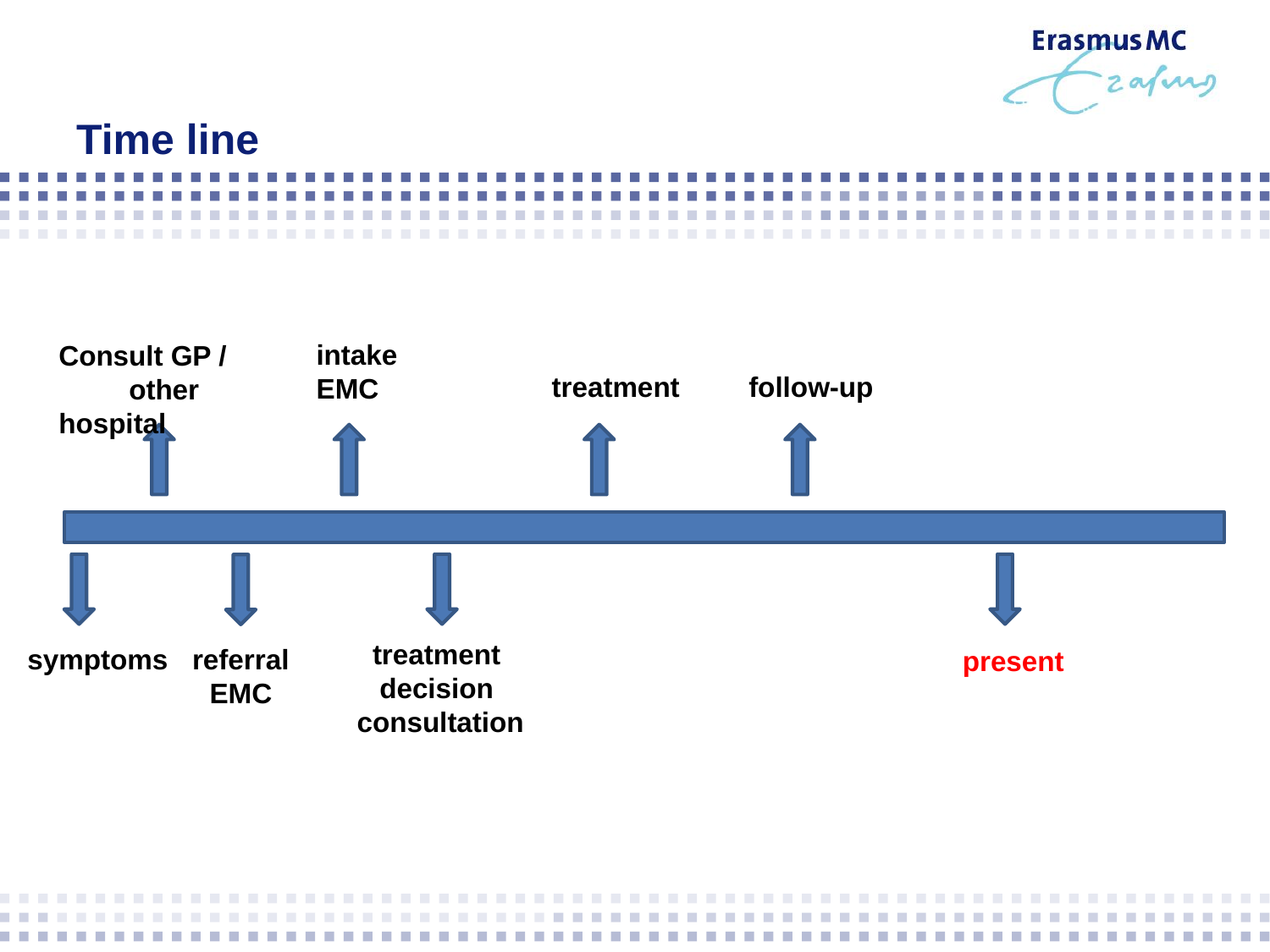

# Time line
intake
EMC
Consult GP / other hospital
treatment
follow-up
treatment
decision
consultation
symptoms
referral
EMC
present

## Slide 5
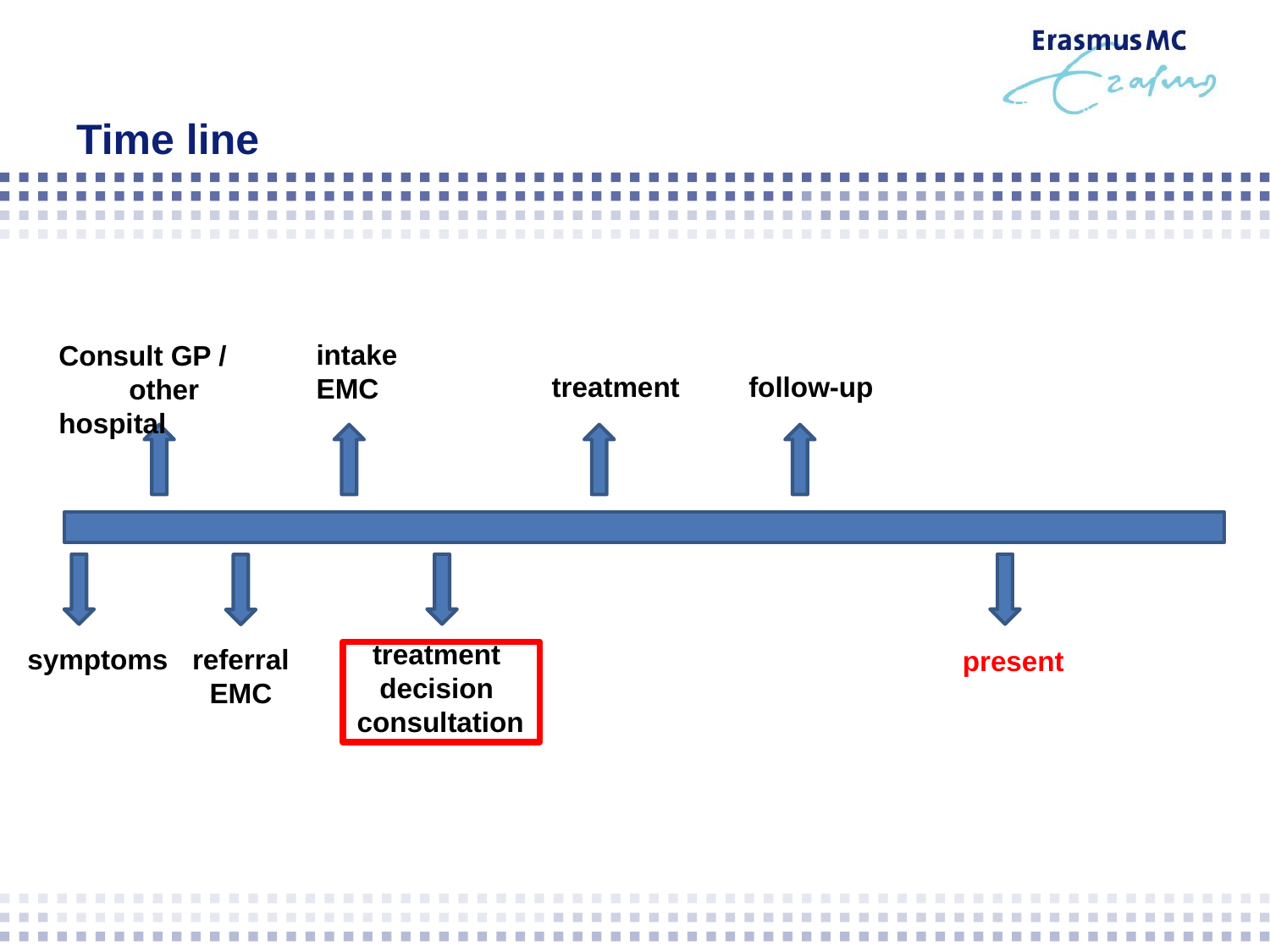

# Time line
intake
EMC
Consult GP / other hospital
treatment
follow-up
treatment
decision
consultation
symptoms
referral
EMC
present

## Slide 6
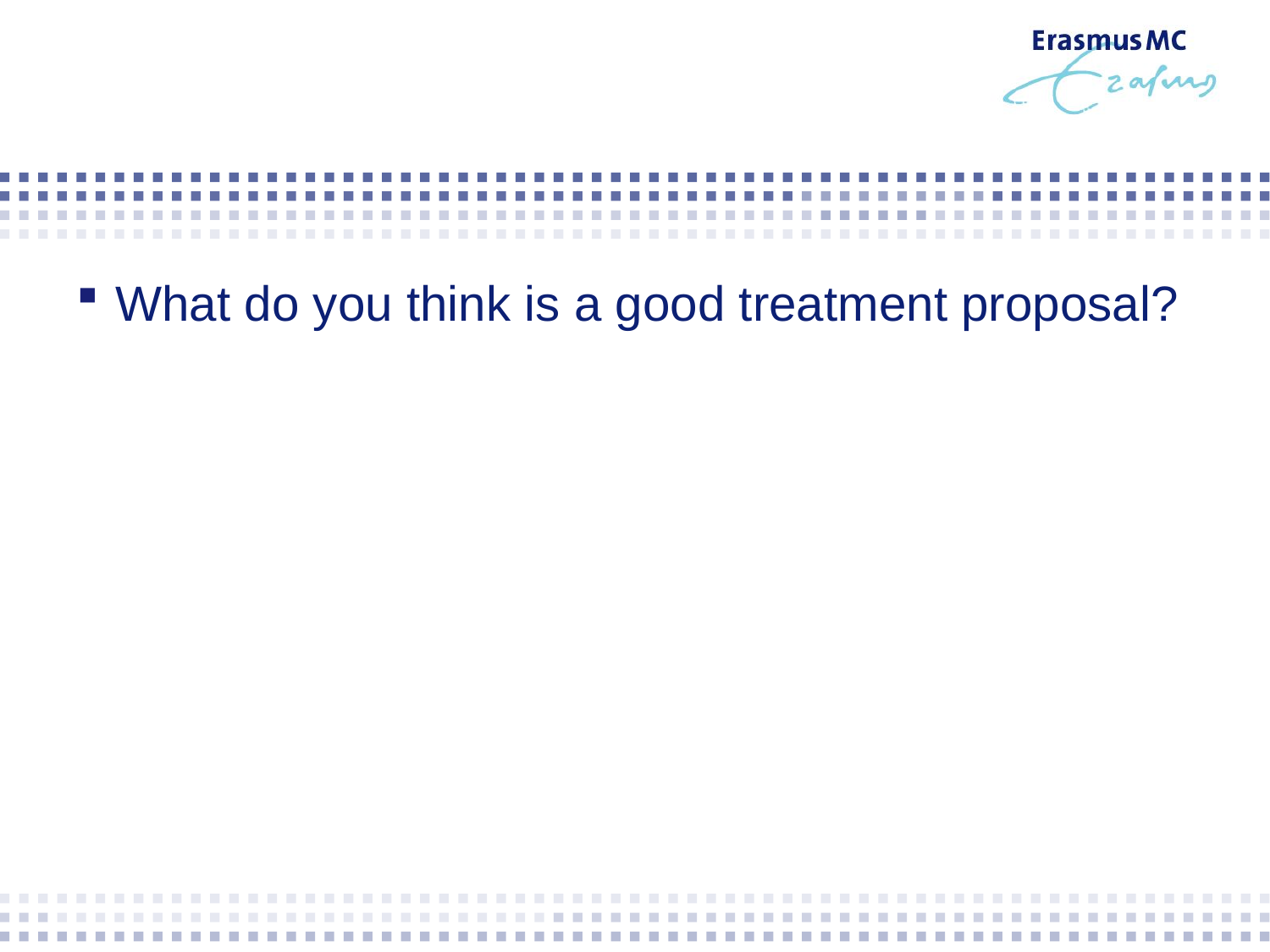

#
What do you think is a good treatment proposal?

## Slide 7
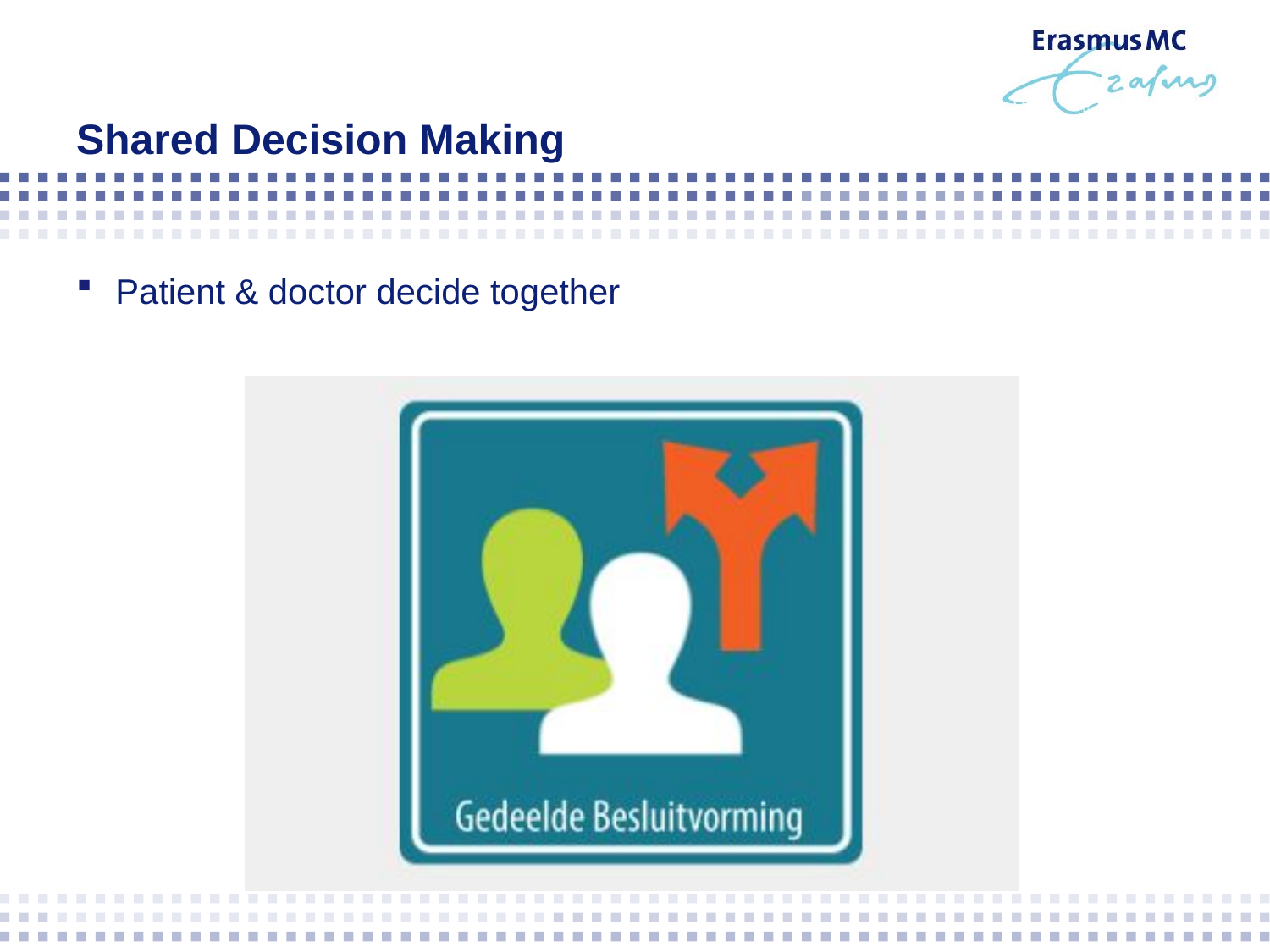

# Shared Decision Making
Patient & doctor decide together

## Slide 8
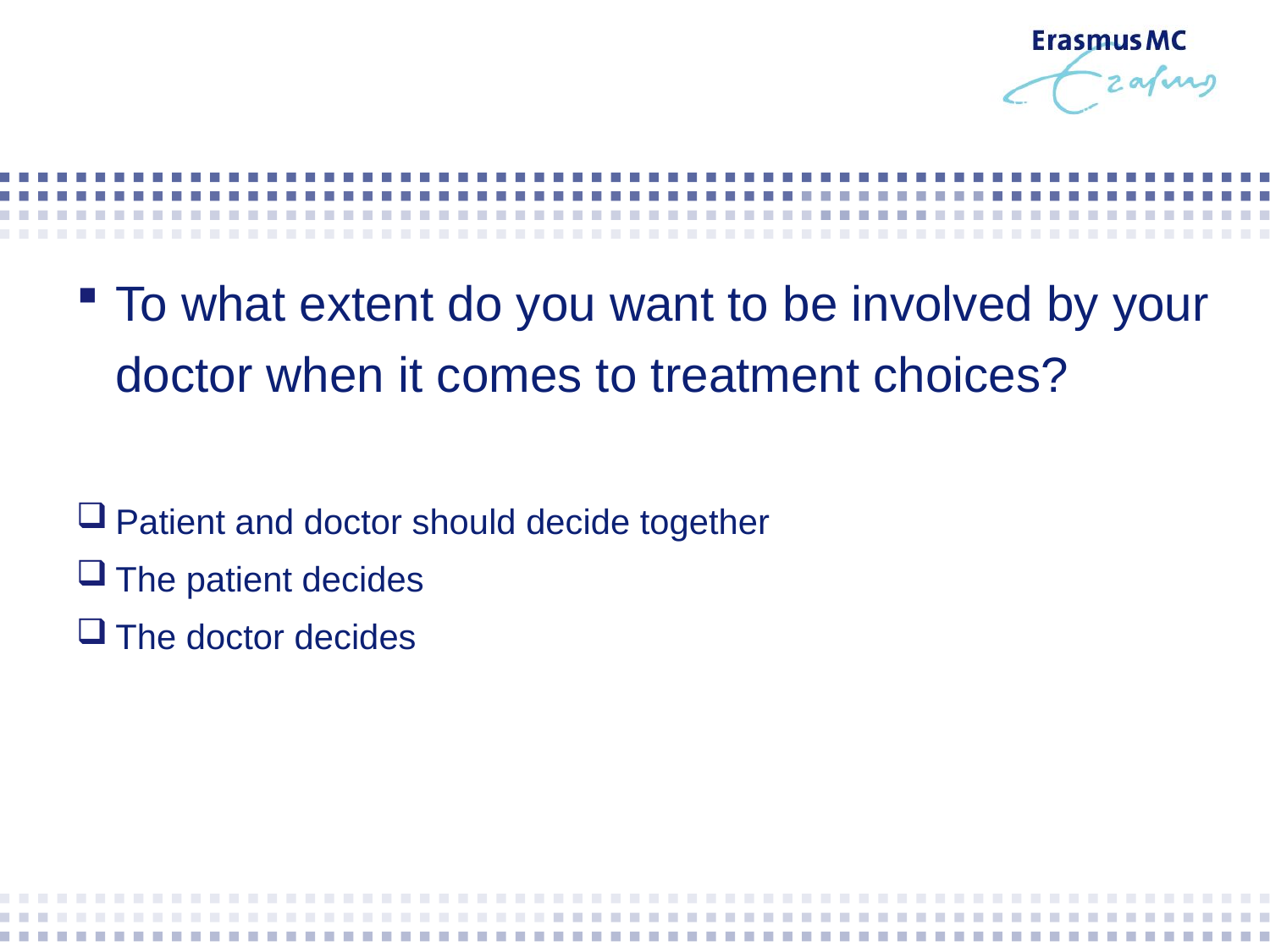

#
To what extent do you want to be involved by your doctor when it comes to treatment choices?
Patient and doctor should decide together
The patient decides
The doctor decides

## Slide 9
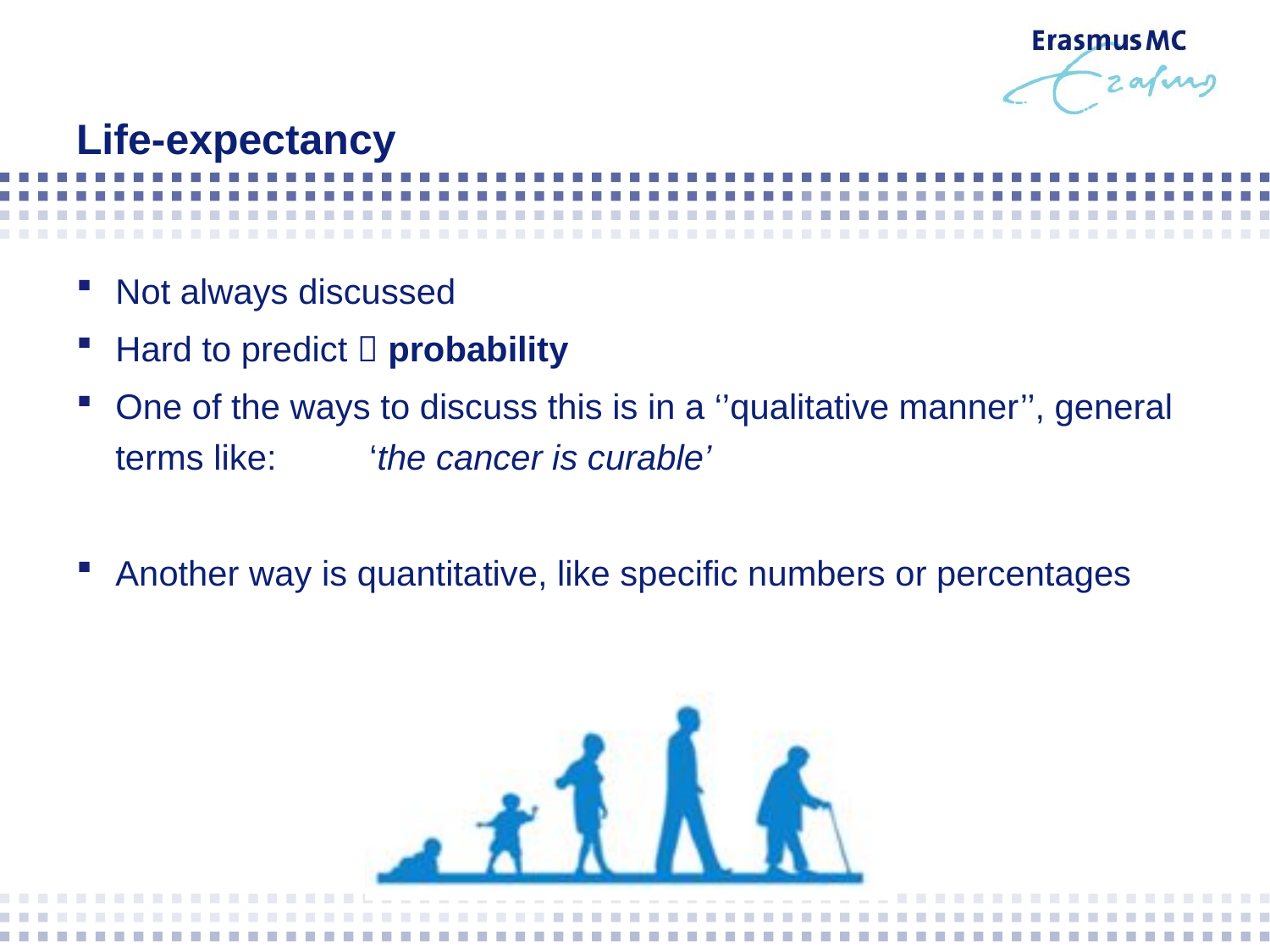

# Life-expectancy
Not always discussed
Hard to predict  probability
One of the ways to discuss this is in a ‘’qualitative manner’’, general terms like:	‘the cancer is curable’
Another way is quantitative, like specific numbers or percentages

## Slide 10
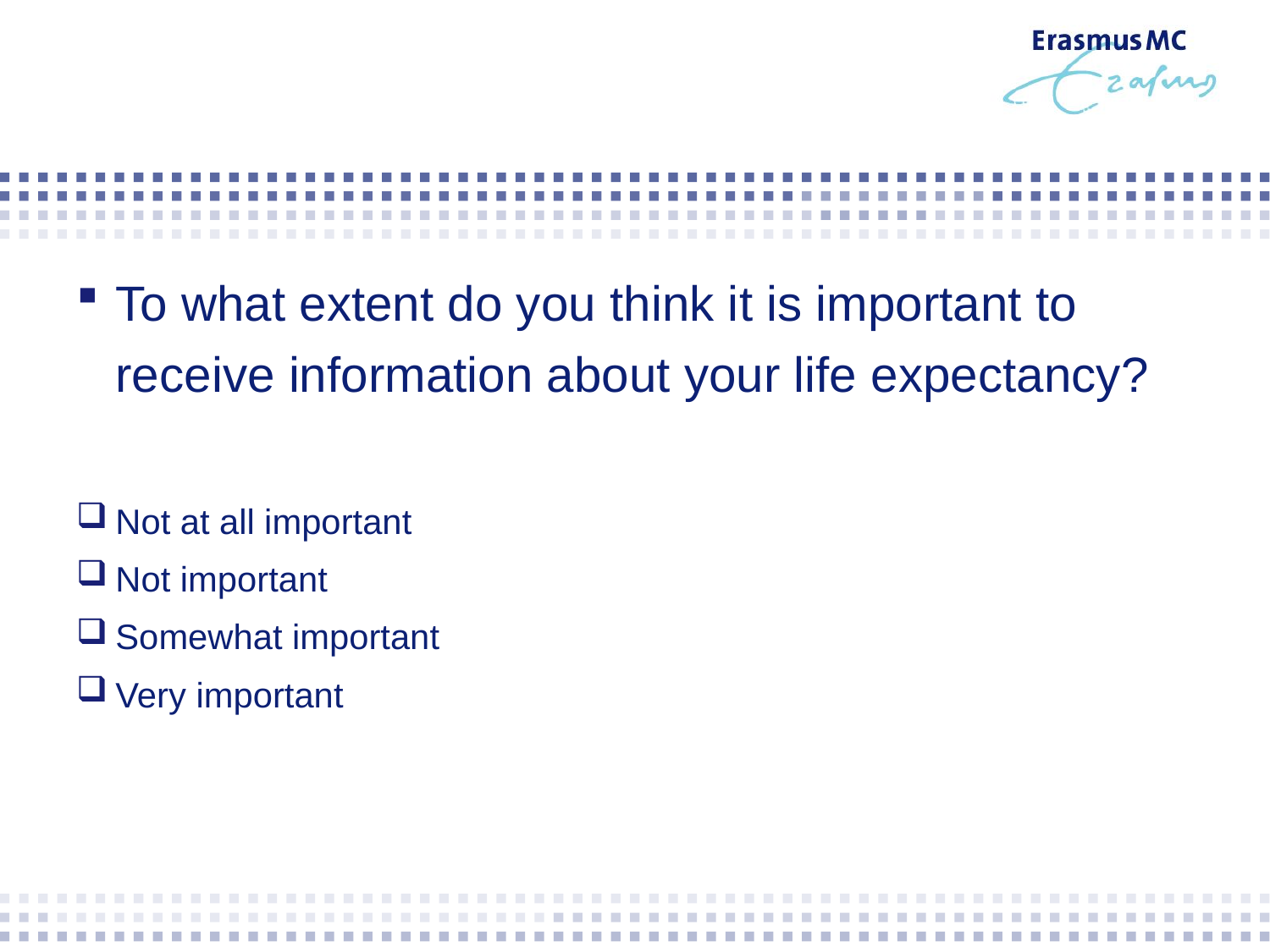

#
To what extent do you think it is important to receive information about your life expectancy?
Not at all important
Not important
Somewhat important
Very important

## Slide 11
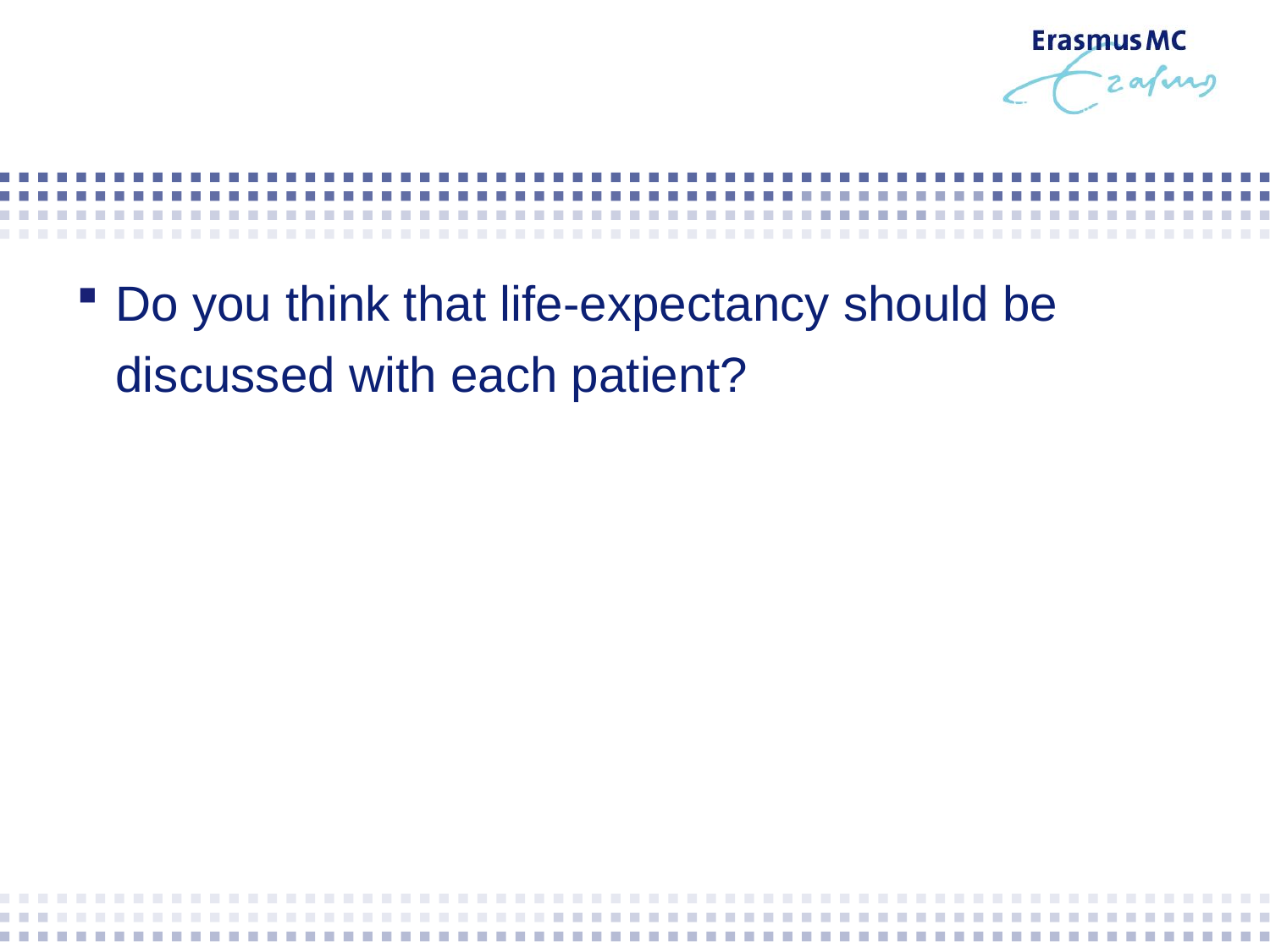

#
Do you think that life-expectancy should be discussed with each patient?

## Slide 12
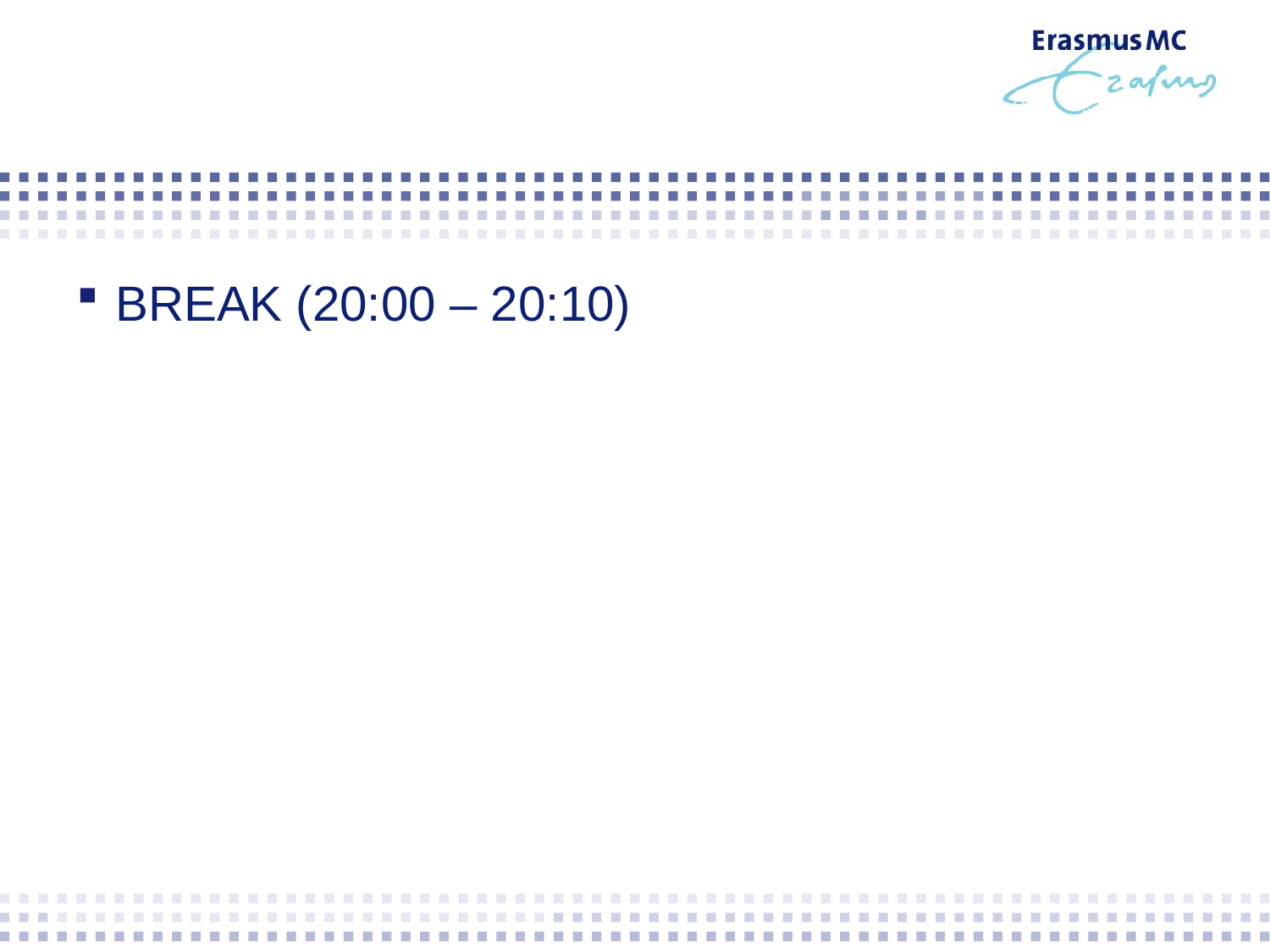

#
BREAK (20:00 – 20:10)

## Slide 13
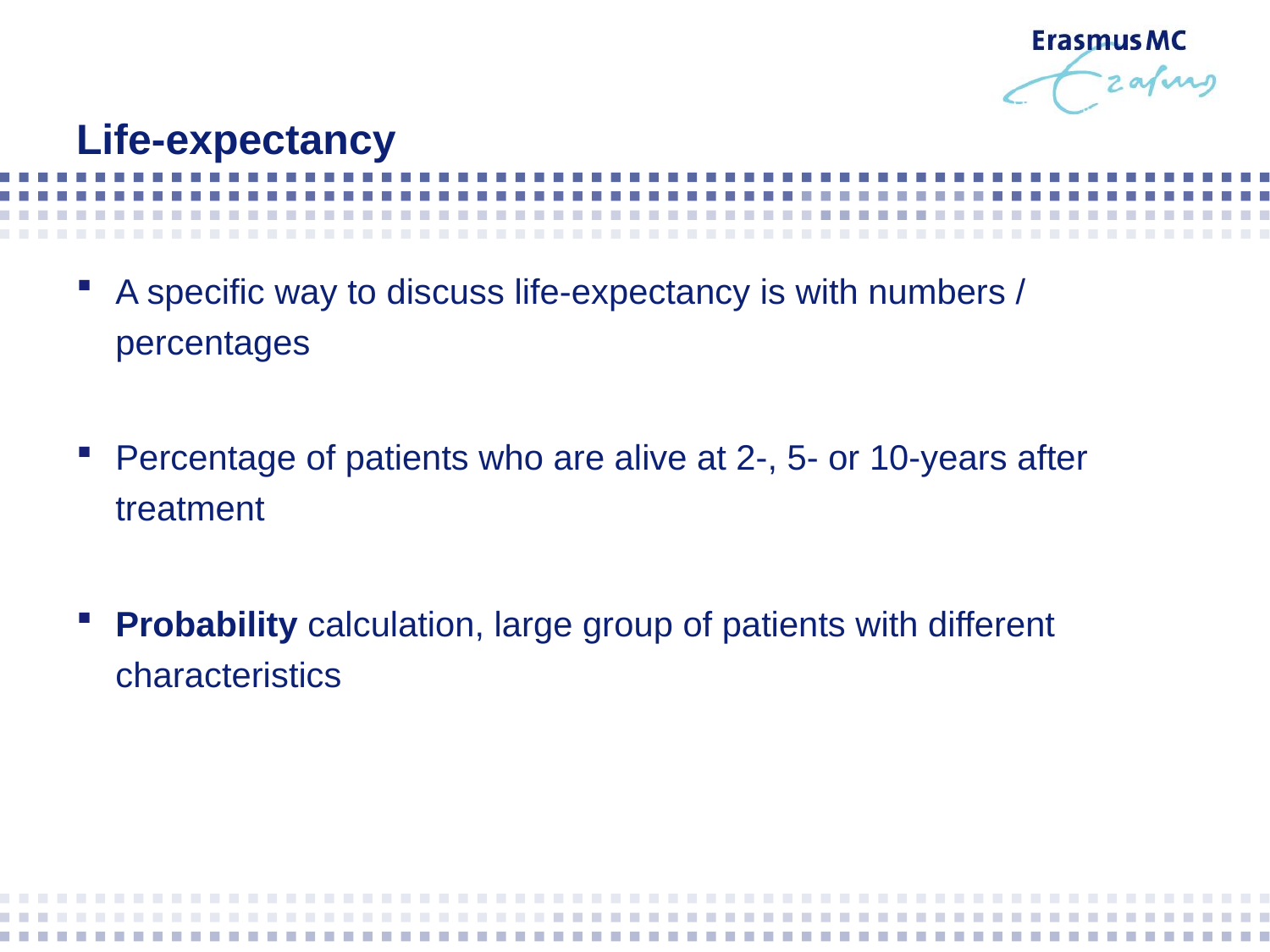

# Life-expectancy
A specific way to discuss life-expectancy is with numbers / percentages
Percentage of patients who are alive at 2-, 5- or 10-years after treatment
Probability calculation, large group of patients with different characteristics

## Slide 14
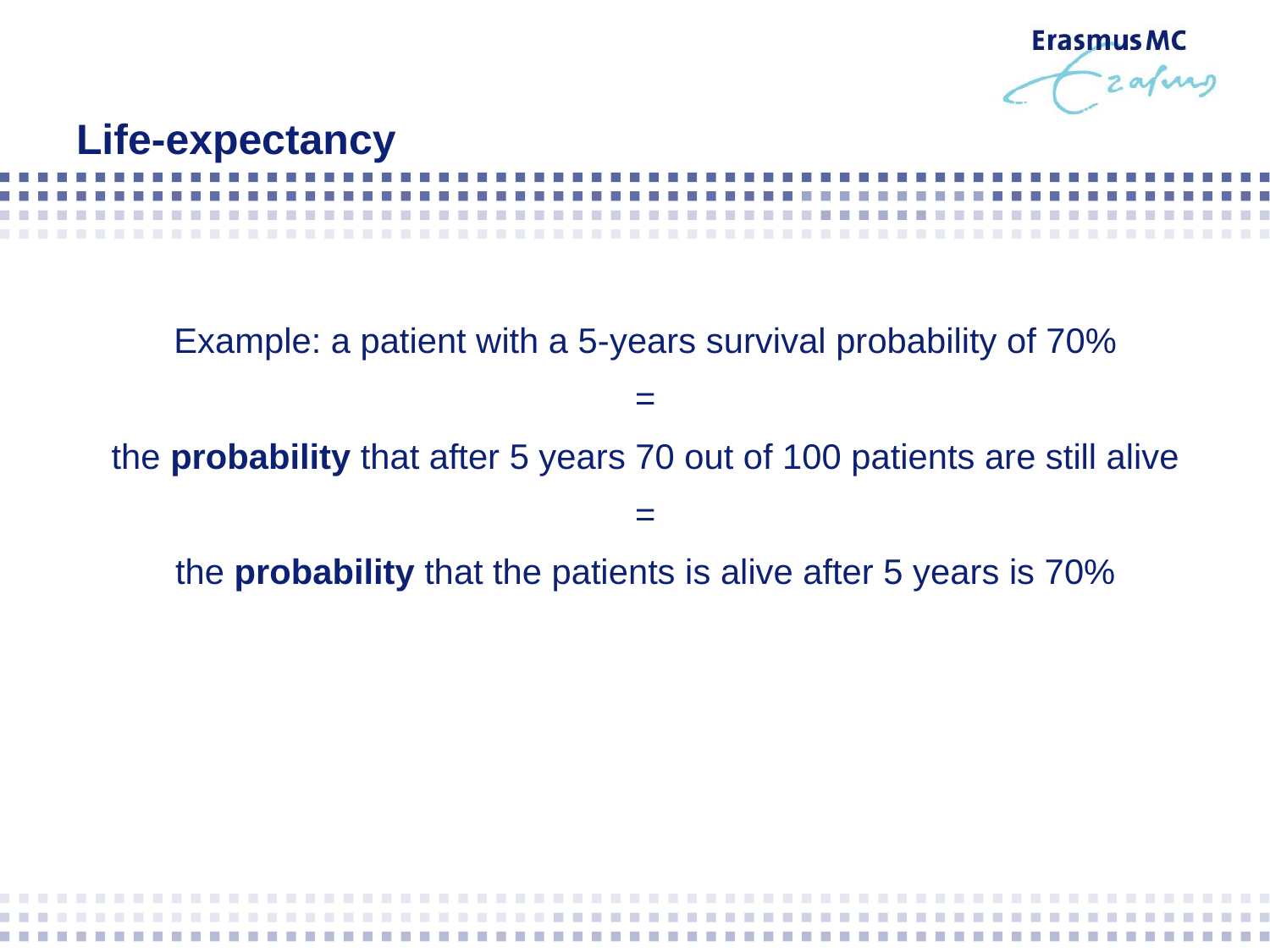

# Life-expectancy
Example: a patient with a 5-years survival probability of 70%
=
the probability that after 5 years 70 out of 100 patients are still alive
=
the probability that the patients is alive after 5 years is 70%

## Slide 15
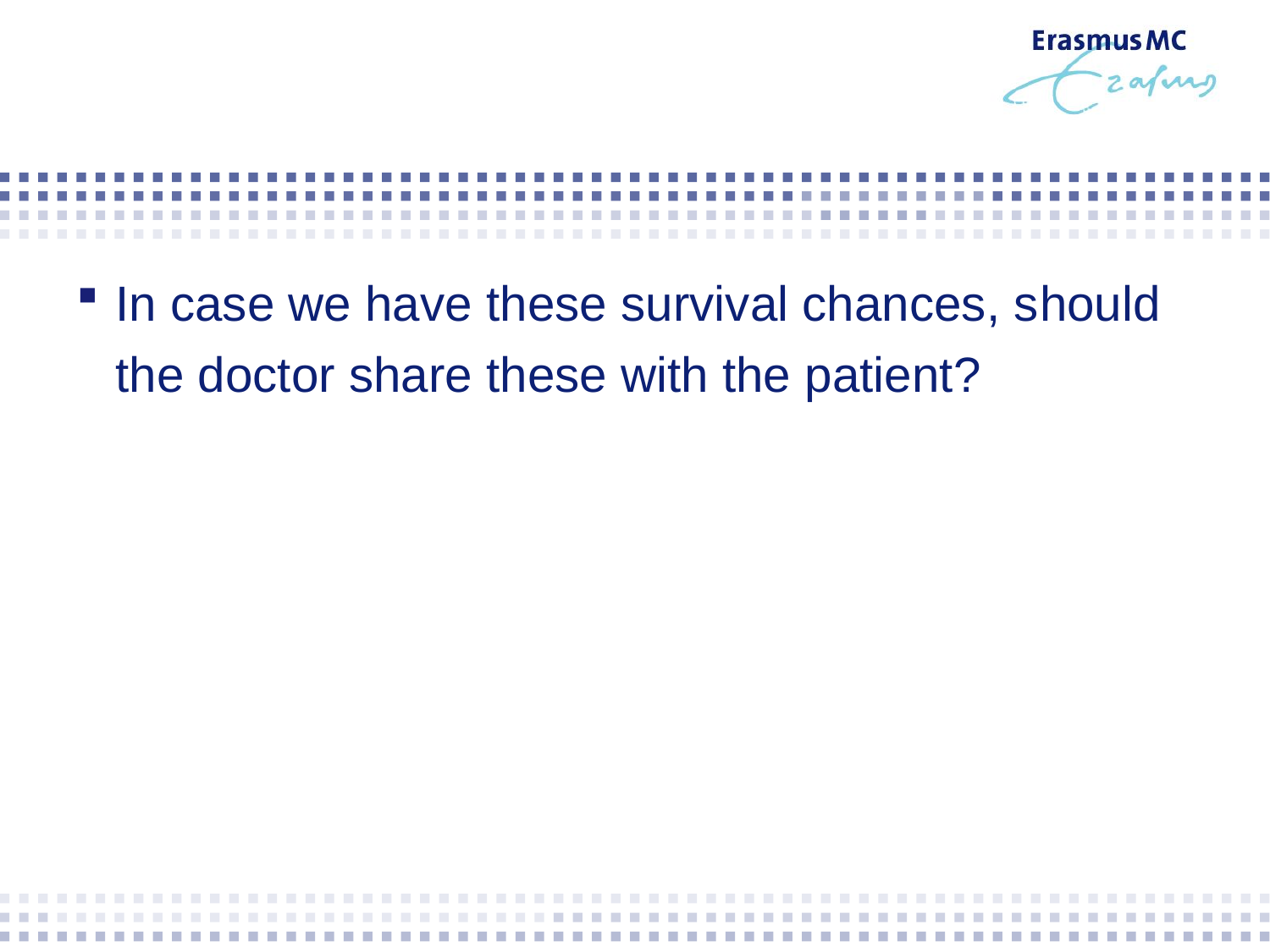

#
In case we have these survival chances, should the doctor share these with the patient?

## Slide 16
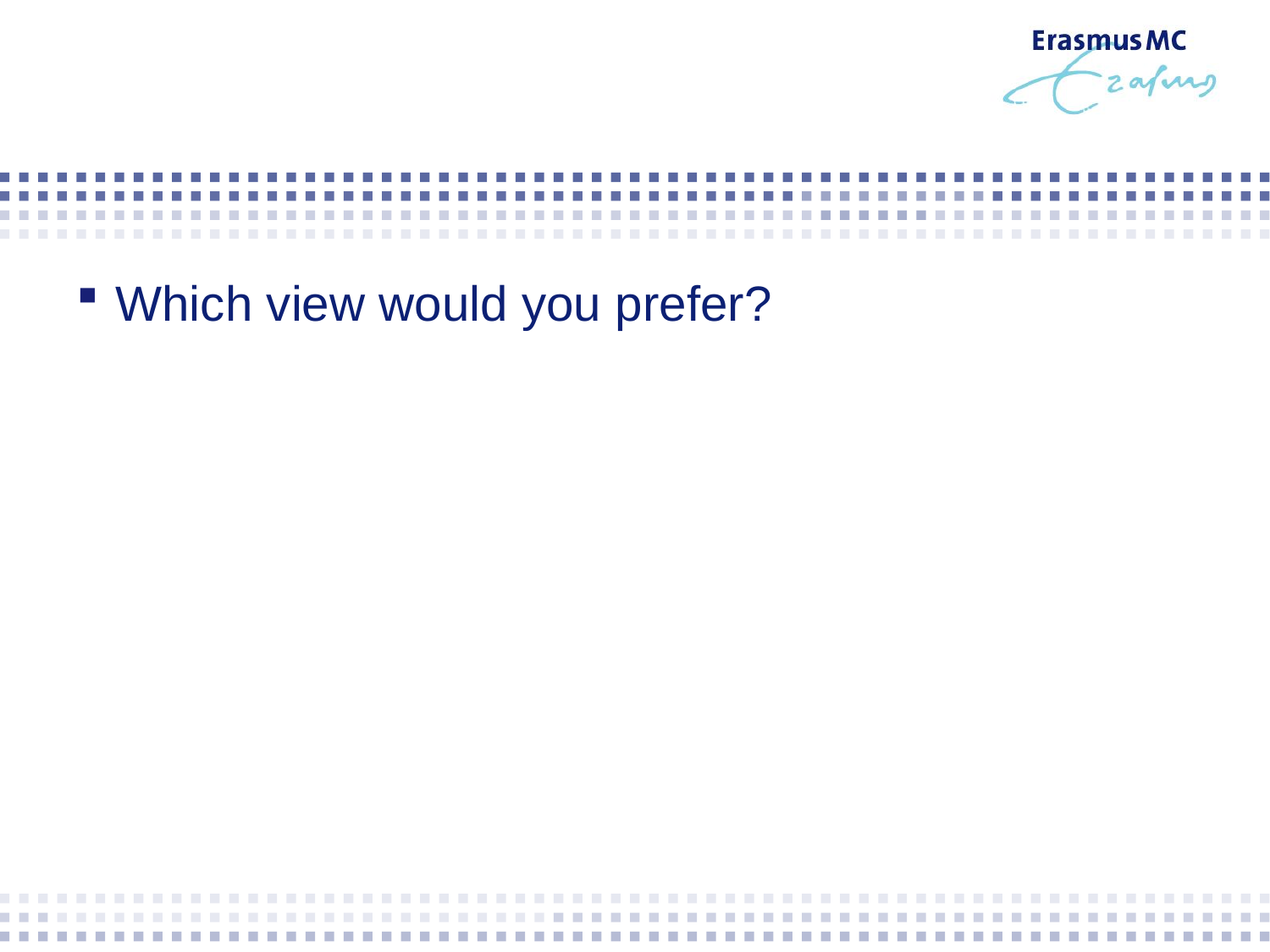

#
Which view would you prefer?

## Slide 17
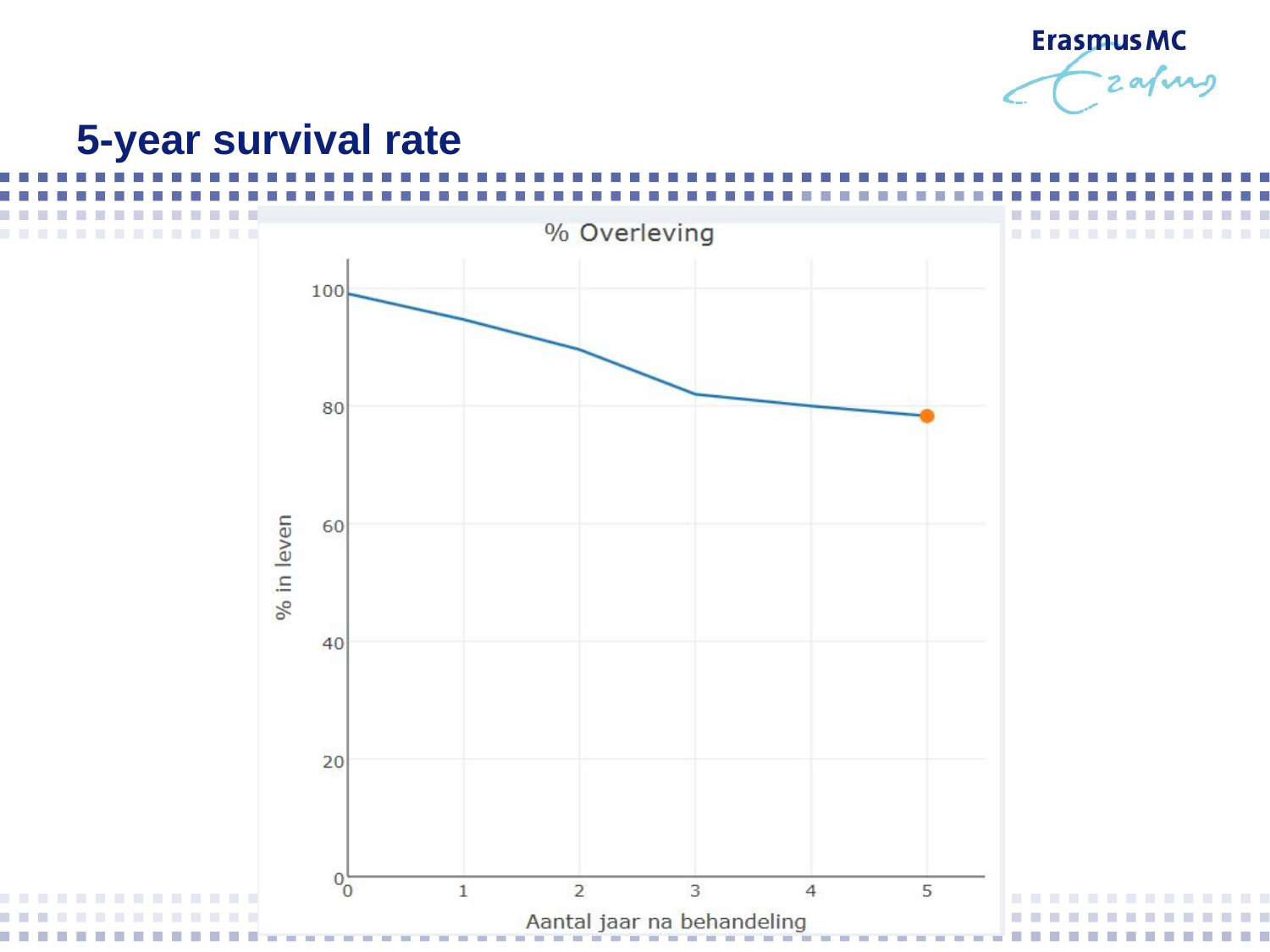

# 5-year survival rate

## Slide 18
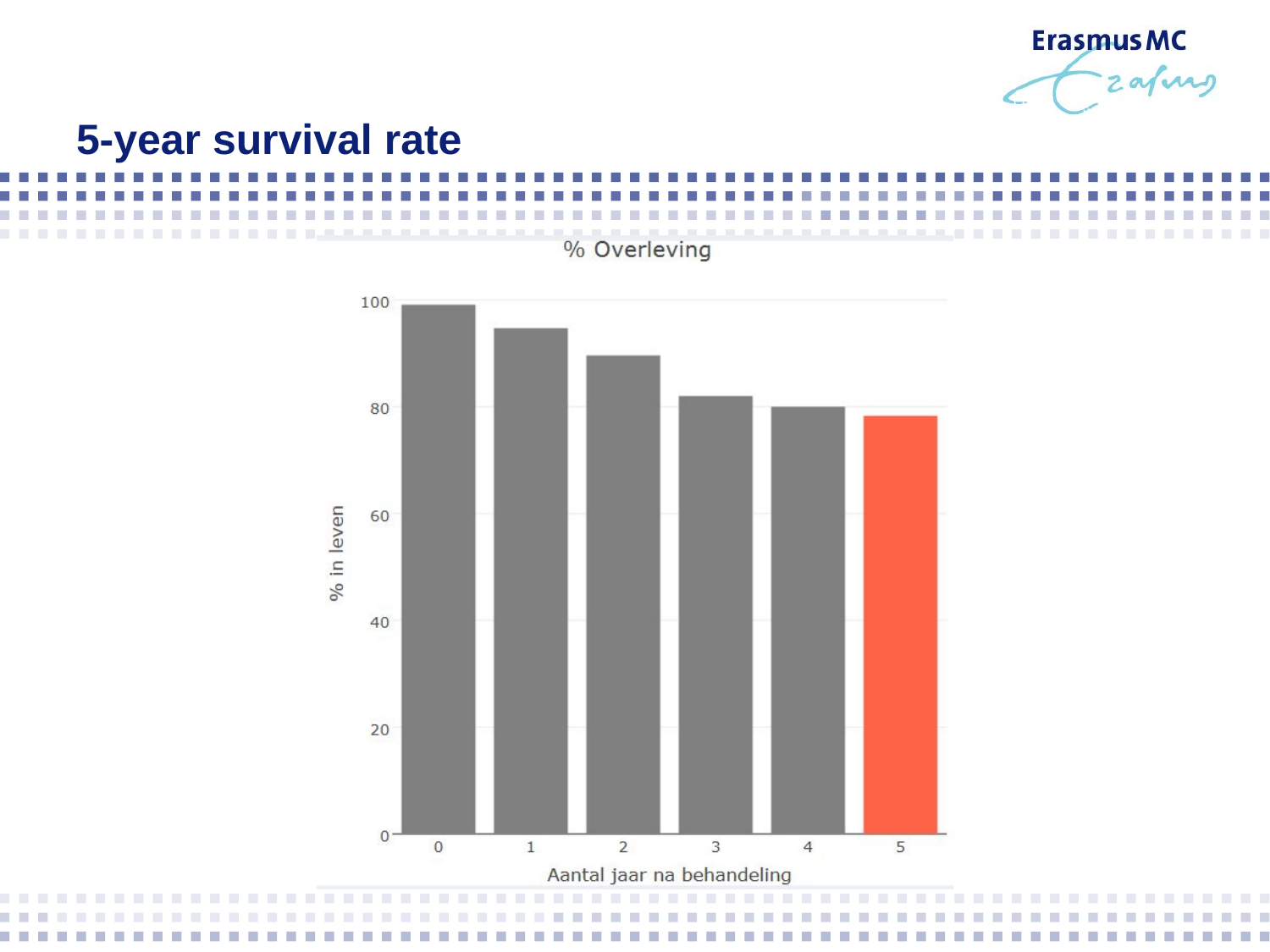

# 5-year survival rate

## Slide 19
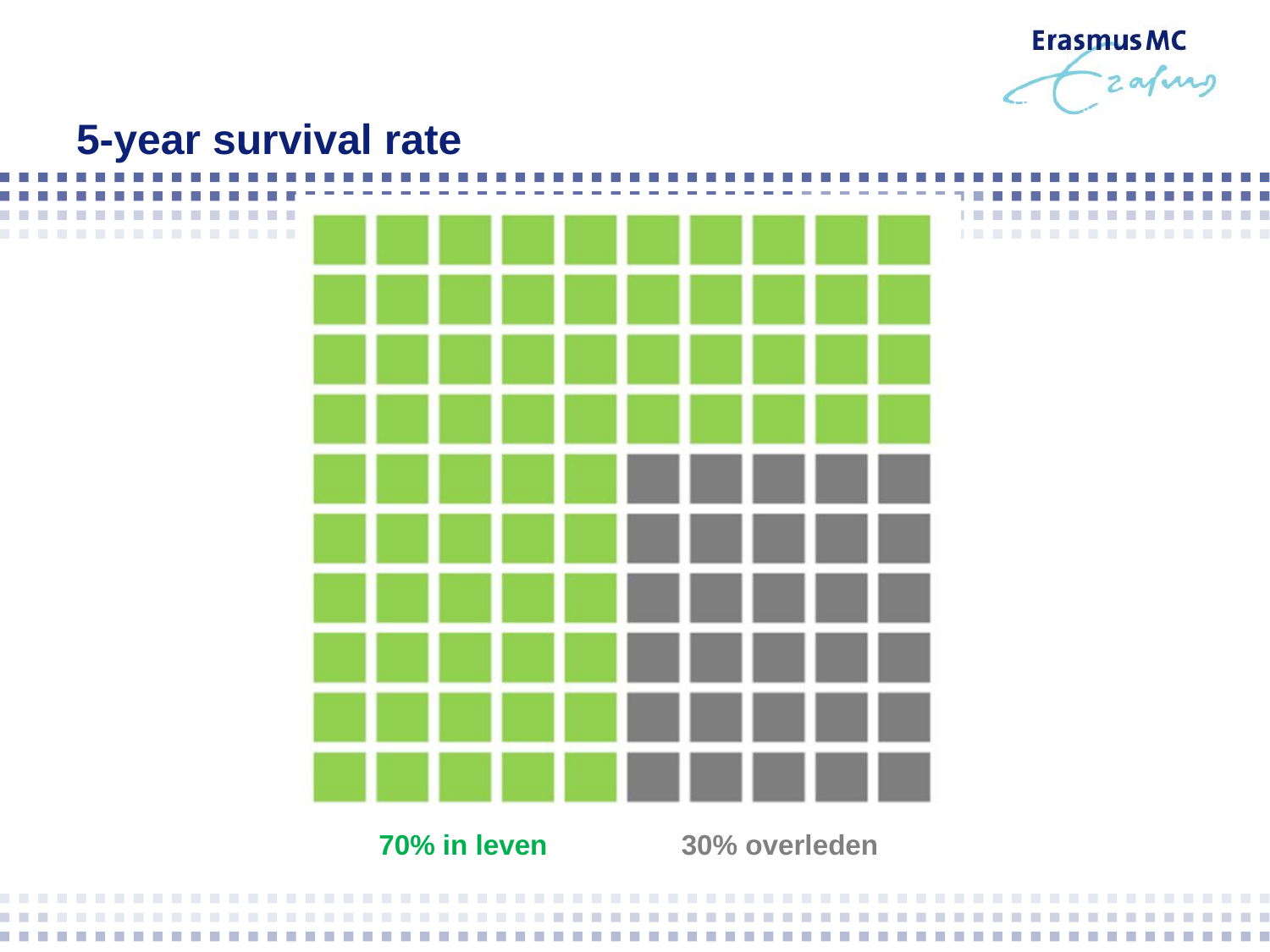

# 5-year survival rate
 70% in leven		30% overleden

## Slide 20
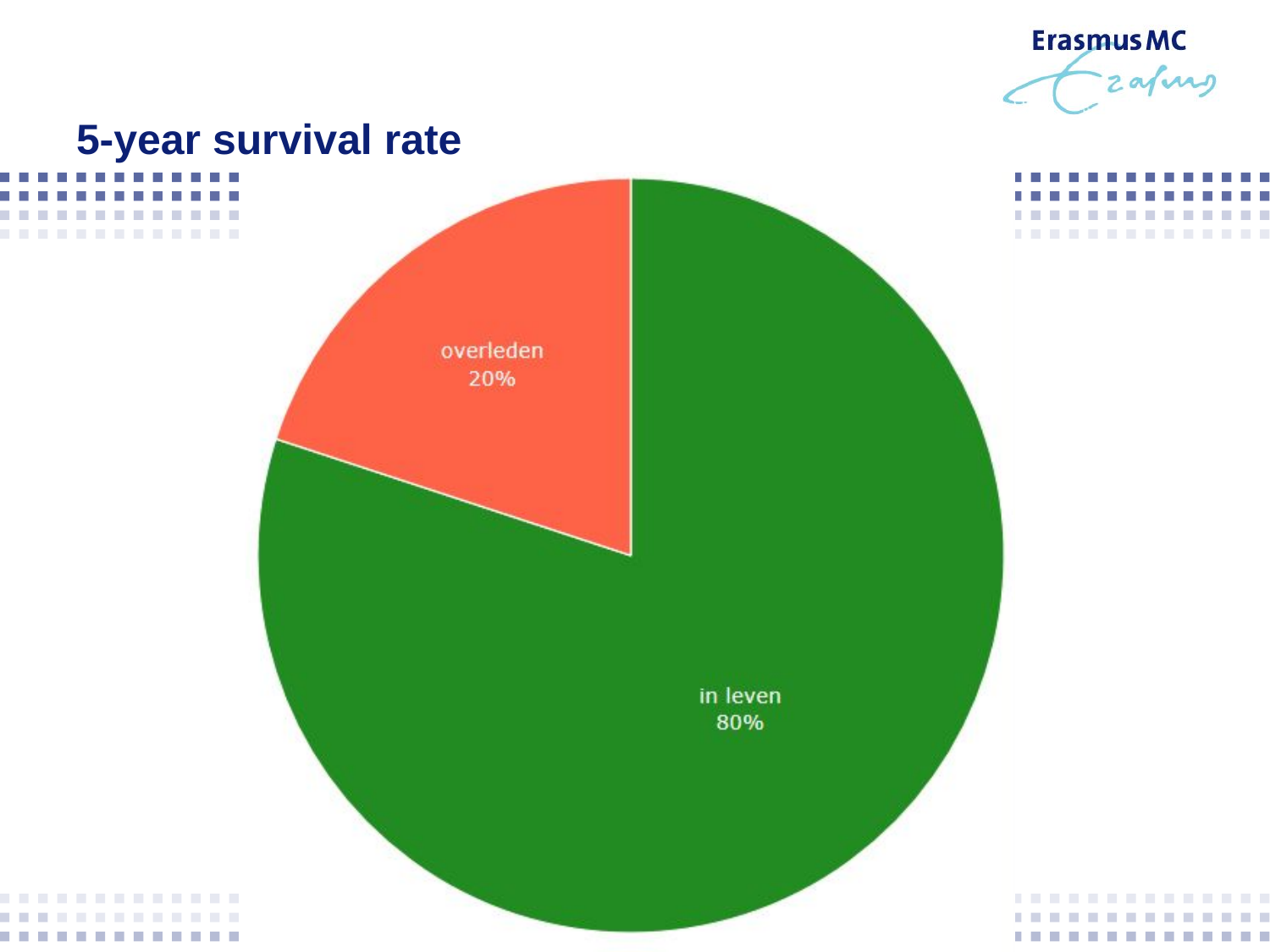

# 5-year survival rate

## Slide 21
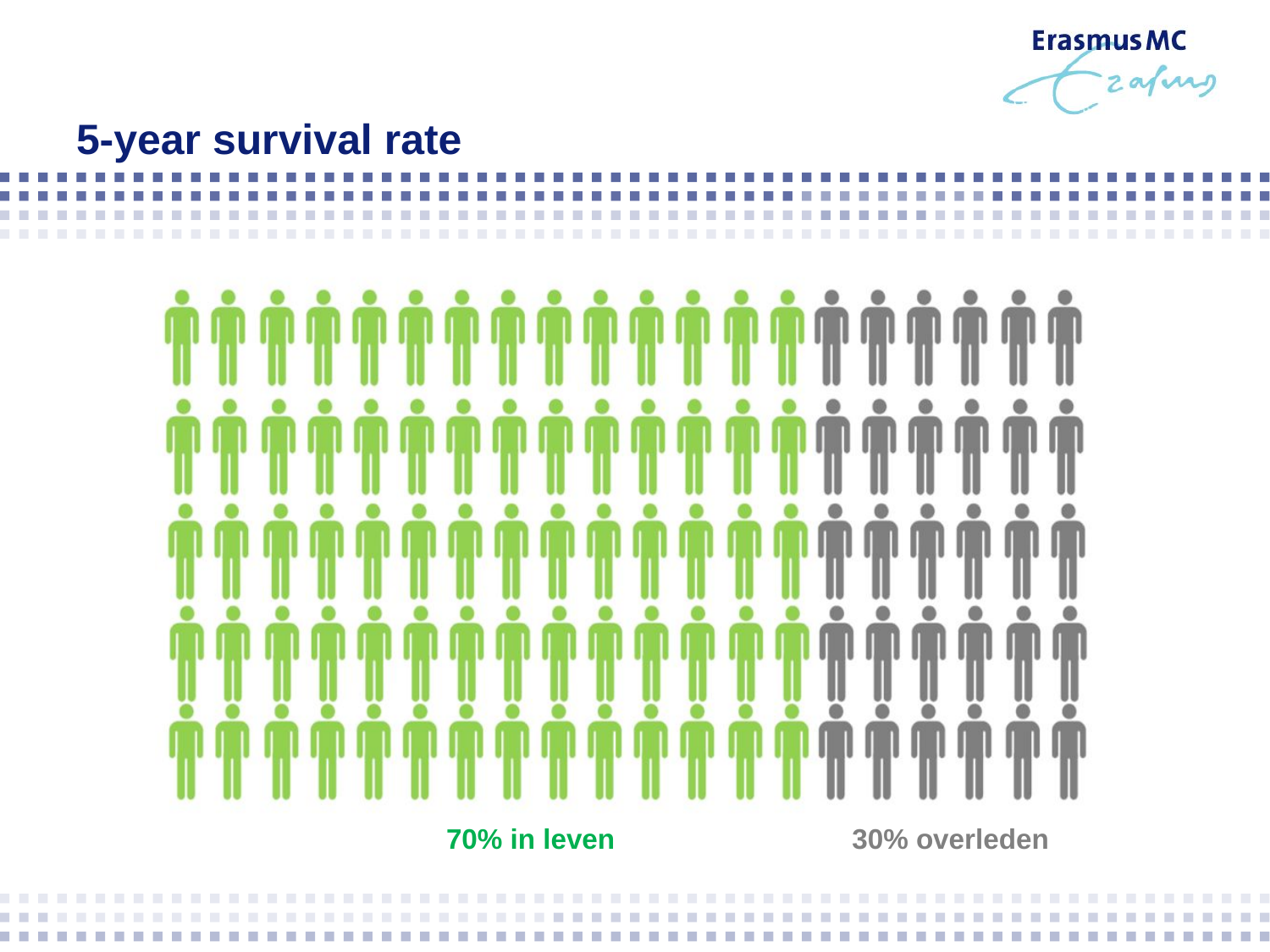

# 5-year survival rate
	 70% in leven	 30% overleden

## Slide 22
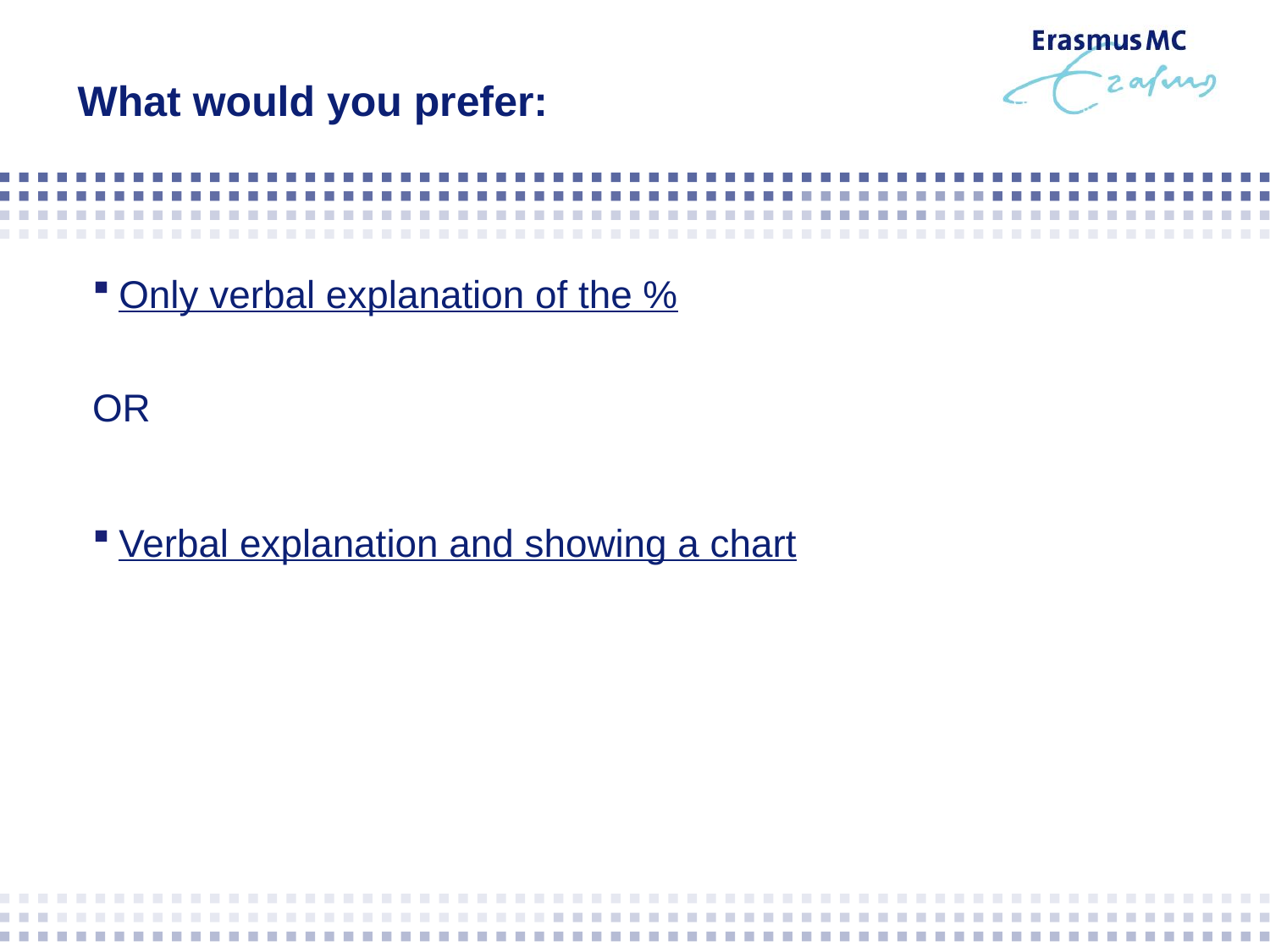

# What would you prefer:
Only verbal explanation of the %
OR
Verbal explanation and showing a chart

## Slide 23
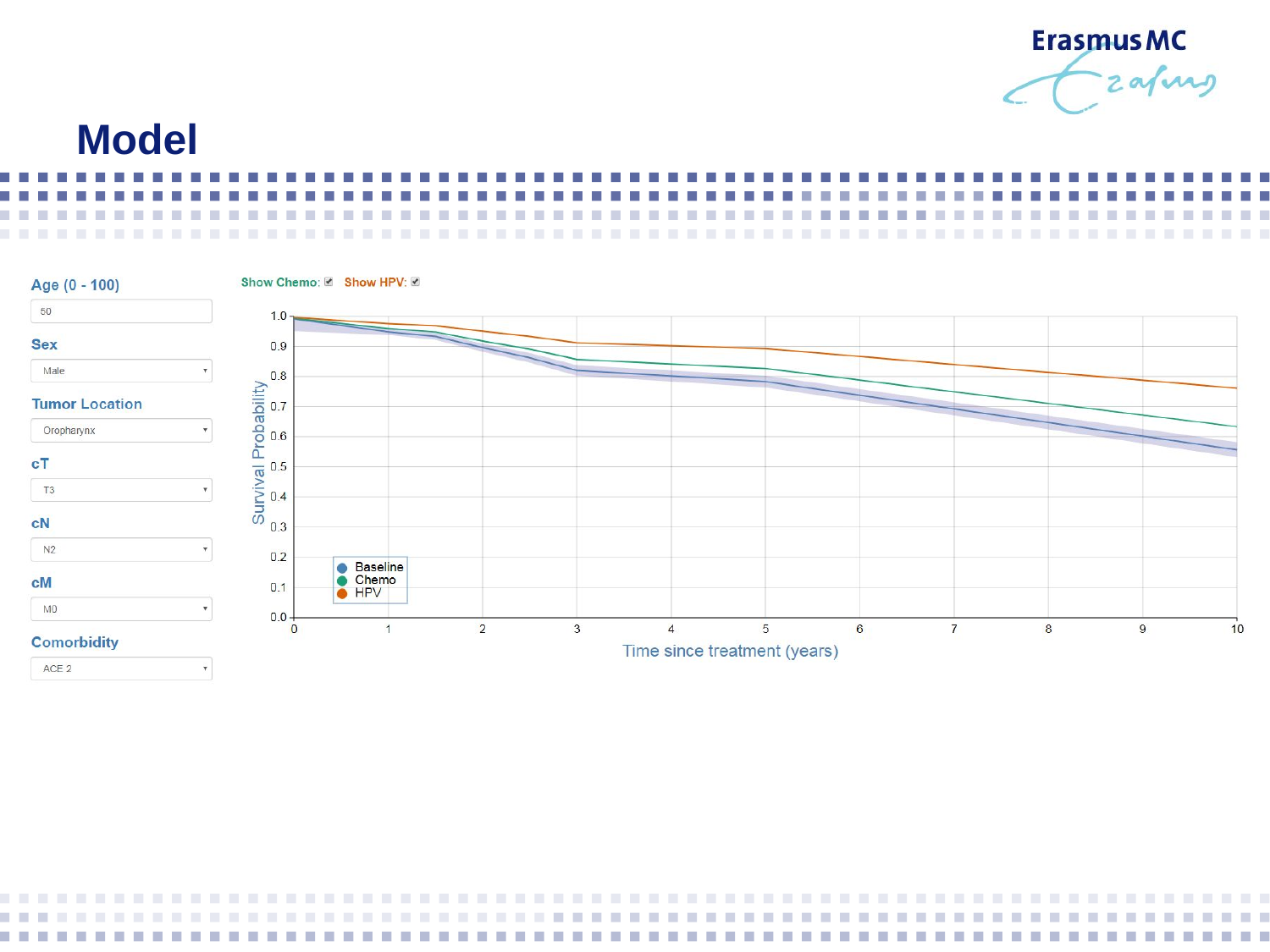

# Model

## Slide 24
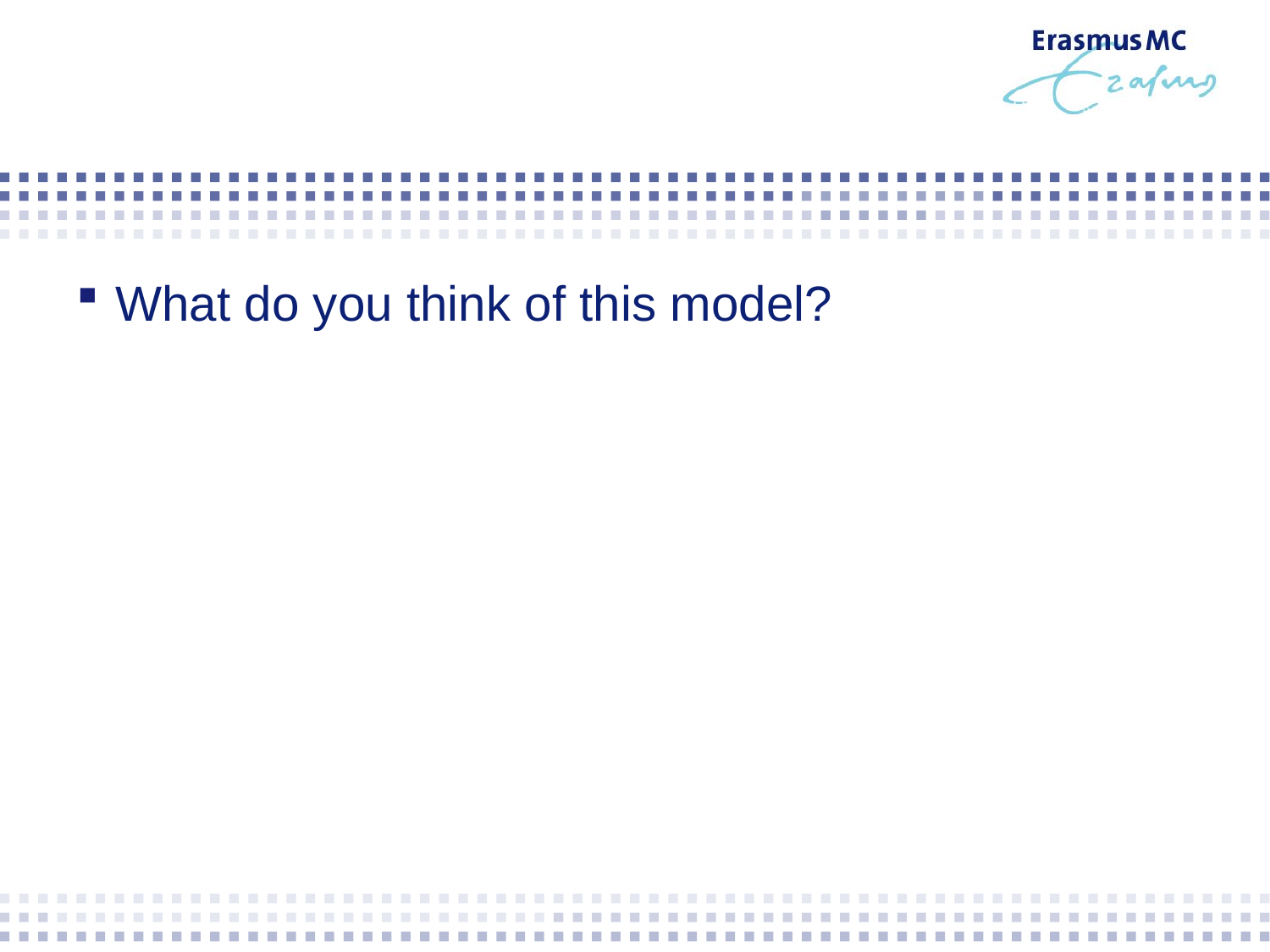

#
What do you think of this model?

## Slide 25
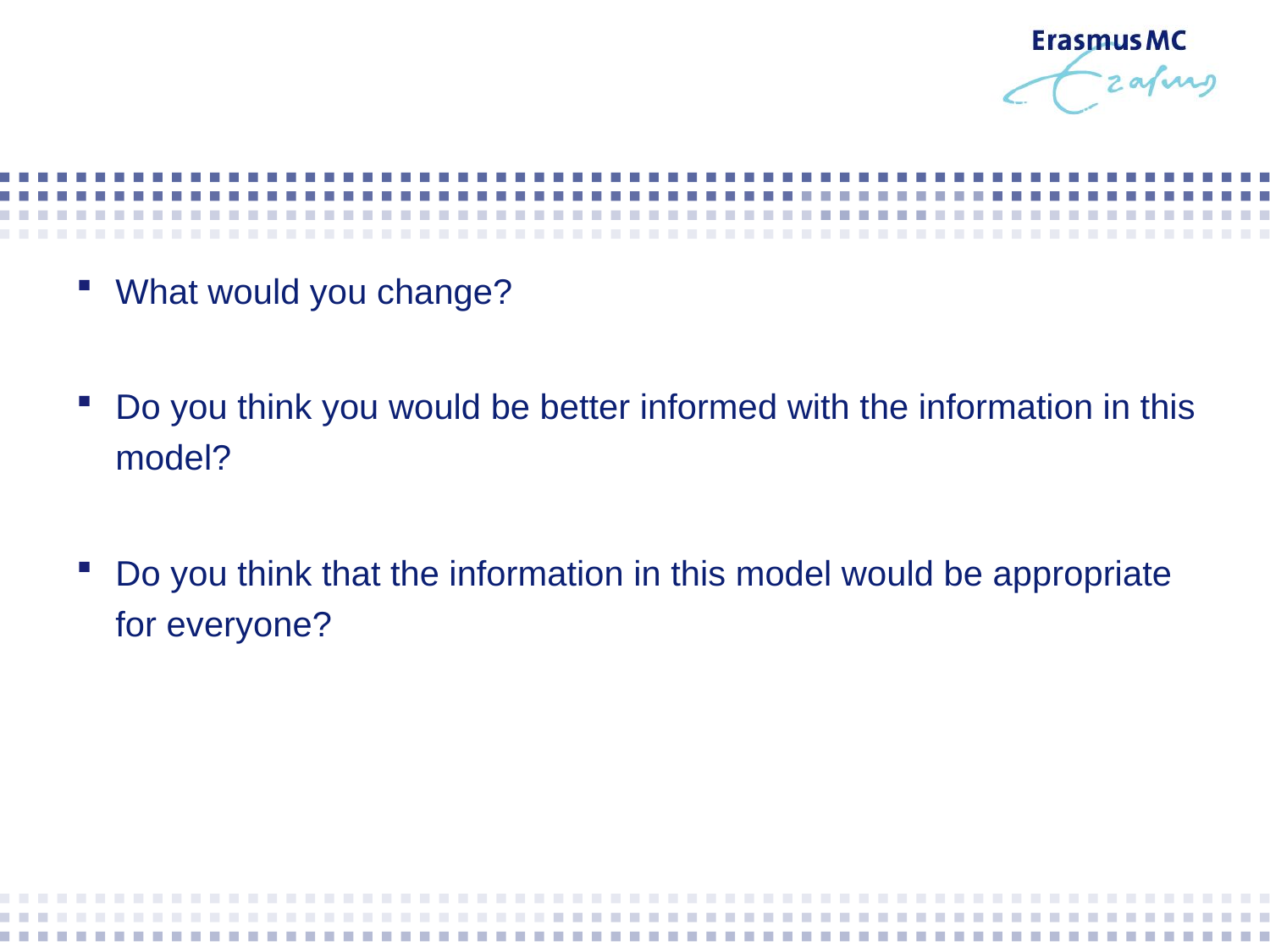

#
What would you change?
Do you think you would be better informed with the information in this model?
Do you think that the information in this model would be appropriate for everyone?
